# A New Saurichthyiform (Actinopterygii) with a Crushing Feeding Mechanism from the Middle Triassic of Guizhou (China)

**DOI:** 10.1371/journal.pone.0081010

**Published:** 2013-12-04

**Authors:** Feixiang Wu, Mee-mann Chang, Yuanlin Sun, Guanghui Xu

**Affiliations:** 1 Key Laboratory of Vertebrate Evolution and Human Origins of Chinese Academy of Sciences, Institute of Vertebrate Paleontology and Paleoanthropology, Chinese Academy of Sciences, Beijing, China; 2 Key Laboratory of Orogenic Belts and Crustal Evolution, School of Earth and Space Sciences, Peking University, Beijing, China; Team ‘Evo-Devo of Vertebrate Dentition’, France

## Abstract

**Background:**

Equipped with an effective predatory feeding mechanism enhanced by large and sharp teeth, pointed snout and elongate body, saurichthyiform fishes are considered common fish-eaters in the early Mesozoic aquatic ecosystems. Additionally, because of the similar body plan across species, saurichthyiforms are also regarded evolutionally conservative, with few morphological and ecological changes during their long history. However, their phylogenetic affinity remains unclear as to whether they are chondrostean, neopterygian or stem-actinopteran, and likewise the intrarelationships of the group have rarely been explored.

**Methodology/Principal Findings:**

Here we report a new saurichthyiform from the Middle Triassic of Guizhou, China, based on the well-preserved specimens including a 3-D braincase. The new taxon, *Yelangichthys macrocephalus* gen. et sp. nov., is unique among saurichthyiforms in having a peculiar neurocranium with a broad orbital tectum, paired posterior myodomes, a deep, transverse fossa in the posterodorsal part of the orbit, and a feeding mechanism structured for durophagy. Phylogenetic analysis places *Yelangichthys* gen. nov. at the most basal position in the Saurichthyiformes as the sister to Saurichthyidae, and a new family Yelangichthyidae is erected to include only *Y. macrocephalus* gen. et sp. nov. The monophyly of the Chondrostei comprising [Saurichthyiformes + Acipenseriformes] Birgeriiformes is supported, but not the monophyly of *Saurichthys*, the type genus of Saurichthyidae. With its outstanding osteological details, *Yelangichthys* gen. nov. greatly increases the neurocranial variations in saurichthyiforms, and its novel feeding structure suggests the consumption of hard-preys instead of fishes.

**Conclusions/Significance:**

Our findings highlight the detailed osteology of a saurichthyiform braincase and its feeding design. We suggest that saurichthyiforms are closely allied to the Acipenseriformes. Saurichthyiforms were very diverse in the cranial osteology and they might have undergone a rapid evolutionary radiation via, for the new material here, transforming the feeding mechanism and thus exploiting the food resources unsuitable for other saurichthyiforms.

## Introduction

Saurichthyiformes is a morphologically distinct actinopterygian group with an obscure taxonomy and uncertain phylogenetic relationships [1]–[4], whose members range from the Upper Permian (Changhsingian) [5] to the Lower Jurassic and were globally distributed in Triassic [1]–[4], [6]–[9]. Their particular beak-like rostrum, elongated body, opercular apparatus composed only of a single bony gill cover, and abbreviated diphycercal caudal fin make them one of the most specialized fossil group among the lower actinopterygians (non-teleost actinopterygians). However, there have been many debates over their systematic position and interspecific relationships since Agassiz's first report in 1834 [1], [2], [4], [10], even to this day when the study of the lower actinopterygian phylogeny has made great progress thanks to a handful of influential works [11]–[16]. This unsatisfactory situation resulted mostly from the poor quality of the fossil data in general. Most species of the Saurichthyiformes were erected upon incomplete or fragmentary material (for historic review see [1], [2]), even for the type species of *Saurichthys*, the type material is just a fragmentary rostrum [10]. However, a few examples were based on well-preserved and comprehensively described neurocranium, which seems morphologically conservative: there are very few significant neurocranial differences between the Early Triassic and Jurassic forms [1], [17]–[19]. The information gaps had inevitably induced uncertainties in the previous phylogenetic analyses in which *Saurichthys* was exclusively chosen as the terminal taxon. Some selected *Saurichthys ornatus* [1], known mostly by the skull material, for the neurocranial features whereas others picked *S. curionii* for the exoskeletal characters, or assembled the features of both. Obviously, those approaches were far from satisfactory, and thus partly responsible for the ambiguous systematic status of *Saurichthys* in those studies [13]–[16]. Challenges also come from the intrarelationships of saurichthyiforms, for which no attempt has been made since Rieppel's cladistic work on *Saurichthys* in 1992 [4], albeit using rather few (only eight) characters and excluding the Jurassic forms (*Saurorhynchus*). Paradoxically, as the number of species assigned to *Saurichthys* increases because of the morphological diversifications, the taxonomic value of this type genus decreases. Unfortunately, underlying this situation is the still poorly-understood lower actinopterygian phylogeny, of which the satisfactory resolution has been hampered by the limited morphological data, especially the neurocranial ones [13]–[16], [20], [21].

This situation makes the discovery of *Yelangichthys* gen. nov. all the more important because the material consists of a nearly complete cranium showing outstanding anatomical details of the neurocranium. *Yelangichthys* gen. nov. is also important from the functional and ecological perspectives, because it has some peculiar adaptive specializations for a new feeding strategy previously unknown to any saurichthyiform. Additionally, it occurred in Anisian when the saurichthyiform fishes were greatly diversified [3], [22] and the marine ecosystem had run into a fast radiation stage, after recovering from the end-Permian crisis [9], [23], [24]. Mirroring the marine ecosystem of that time, the Panxian-Luoping Fauna in southwestern China contains abundant vertebrates and invertebrates [22], [24]–[28]. Among the fishes, saurichthyiforms are predominant in taxonomical diversity, with at least eight species [22]. As high tier consumers in the trophic pyramid, they were formerly considered as typical carnivores, preying on other smaller fishes or even other saurichthyiform species [2], [4], [19], [29]. However, it is likely not for *Yelangichthys* gen. nov., which displays some structural innovations in the feeding mechanism. Obviously, this new fish will shed new light on further understanding the diversity and evolution of saurichthyiforms.

With this background in mind we aim at four basic objectives in current paper: 1) a detailed description of *Yelangichthys macrocephalus* gen. et sp. nov.; 2) discussion on systematic position of *Yelangichthys* gen. nov.; 3) discussion on interrelationships and intrarelationships of the Saurichthyiformes, as well as testing the monophyly of *Saurichthys*; 4) discussion on specializations of the feeding mechanism of *Yelangichthys* gen. nov. and their ecological and evolutionary significance.

## Materials and Methods

### 1. Geological context

**Figure 1:**
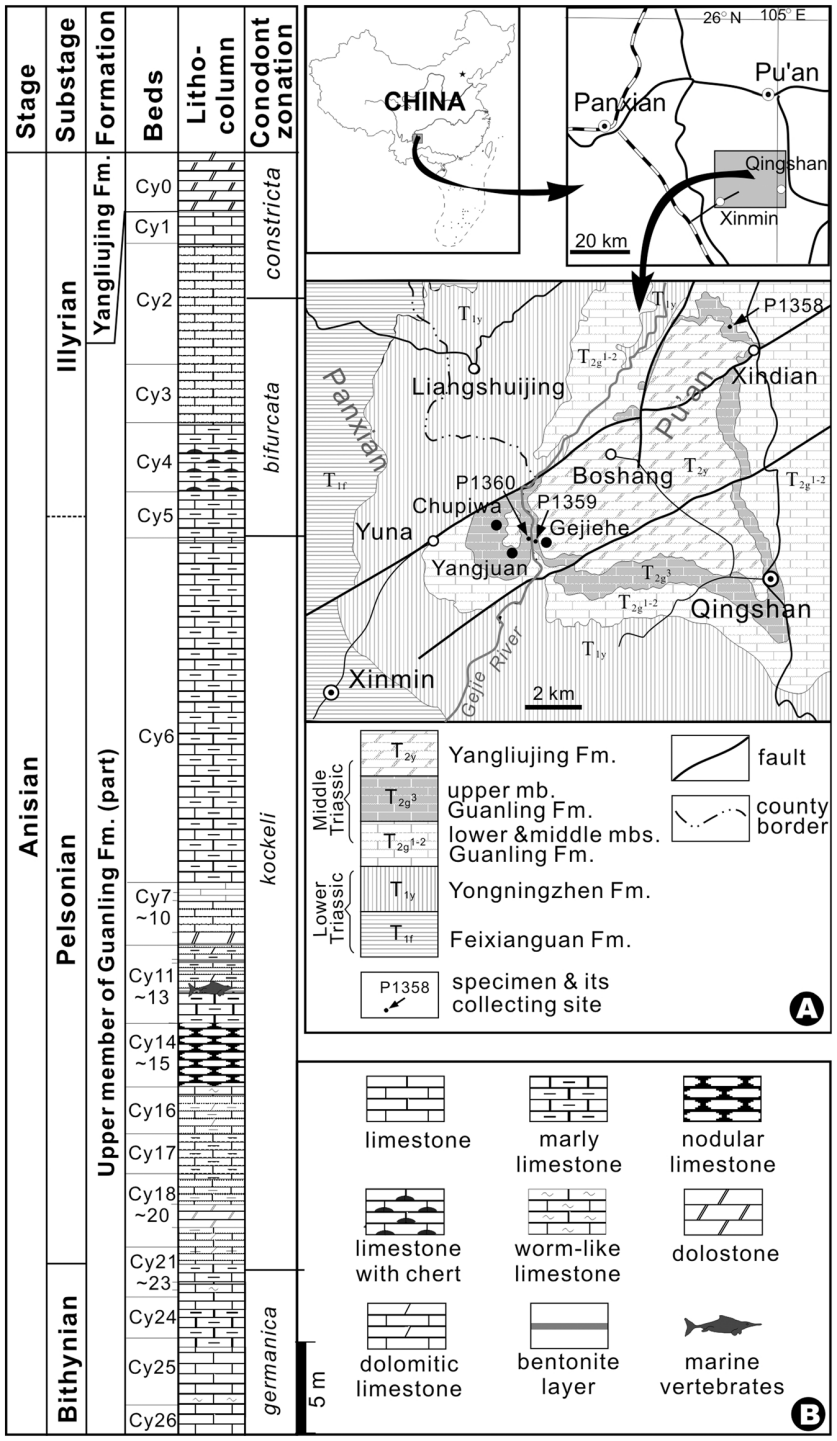
Geological context of *Yelangichthys*. **Left**, lithological column of the fossiliferous strata where the current materials were found in Panxian and Pu' an, Guizhou Province, China. **Right**, (A) geographic and geological map of the locality of fossil materials (P1358-1360); (B) lithological legends.

The study area is on the border between Panxian and Pu'an counties of Guizhou Province, China, where the Lower and Middle Triassic rocks are widely exposed, forming a slightly northeast declined syncline (Fig. 1). The fossil material described here was collected from three sites of the same stratigraphic horizon corresponding to the bed CY-12 in the Upper Member of the Middle Triassic Guanling Formation exposed around Yangjuan and Chupiwa villages (Fig. 1). Abundant marine vertebrate and invertebrate fossils were discovered from the bed CY-12 and the underlying bed CY-13 [3], [22], [27], [30]. The conodont analysis revealed that the fossil-bearing stratum is within the conodont *Nicoraella kockeli* Zone of the middle Anisian [31].

### 2. Materials

The material under study belongs to the Geological Museum of Peking University (GMPKU). It includes three specimens preserved in muddy limestone. One is a well-preserved 3-D braincase (GMPKU-P1358, holotype). Another is a dorsoventrally flattened skull with disarticulated bones of its mandible, opercular series, and dermal shoulder girdle (GMPKU-P1359). The third one is an articulated lower jaw with the anterior tip missing (GMPKU-P1360). The dermal bones are well-preserved in all three specimens, whereas the neurocranium is well preserved in the holotype because the neurocranium is lined with periosteal bone. The description of the dermal bones is based mainly on GMPKU-P1359 and GMPKU-P1360. These specimens, collected from the same strata (Fig. 1), are assigned to the same species for their matching pattern in the structure and ornament of the dermal bones and their identical dentition. The specimens used for comparison are: *Saurichthys dawaziensis* Wu et al., 2009 (GMPKU-P1524) [32]; *Sinosaurichthys longipectoralis* Wu et al., 2011(GMPKU-P1126) [3], *Sinosaurichthys longimedialis* Wu et al., 2011(GMPKU-P1939) [3], *Youngolepis* (IVPP V6234) [33] and *Eosaurichthys chaoi* Liu and Wei, 1988 [5].

The authors have obtained the permission from the Geological Museum of Peking University (GMPKU) to access the collections of the studied material in current paper, and the specimens were collected from the fossil locality by the authors themselves but not purchased, donated, or loaned.

### 3. Methods

Specimens were prepared using air-driven chisel and sharp needles, combined with acid preparation using 10% acetic acid. Most specimens were prepared from both sides. CT-scanning was tried but failed to show the internal anatomy of the neurocranium. Line-drawings were done based on photos, aided by constant examinations of the specimens under a Nikon SMZ1500 binocular. The data matrix was constructed in Winclada [34] and processed using a heuristic search method. We used PAUP v.4.0b10 [35] to run Bremer Support. Character states were treated as equally weighted and unordered.

### 4. Anatomical Nomenclature

Skull bones or structures are generally named following some influential work on *Saurichthys* [1], [2], [4] and other actinopterygians [12], [36], [37], otherwise are given descriptive names, e.g., *vsn* for the network of vermiculate sulci on the ventral side of the orbital tectum.

## Results

### 1. Systematic paleontology

Class OSTEICHTHYES Huxley, 1880

Infraclass ACTINOPTERYGII Cope, 1887

Superorder CHONDROSTEI Müller, 1844

Order SAURICHTHYIFORMES Aldinger, 1937

Family YELANGICHTHYIDAE, fam. nov.

#### Diagnosis

As for the type genus *Yelangichthys*.

#### *Yelangichthys* gen. nov

(Figs. 2–11)

**Figure 2:**
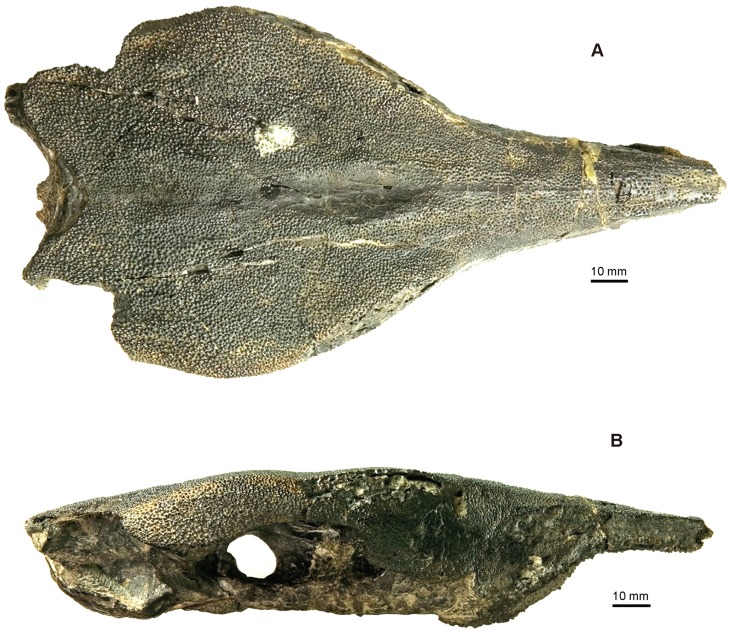
Skull of *Yelangichthys*. Photographs of GMPKU-P1358 (holotype) in **A**, dorsal; **B**, lateral view. Anterior facing right.

**Figure 3:**
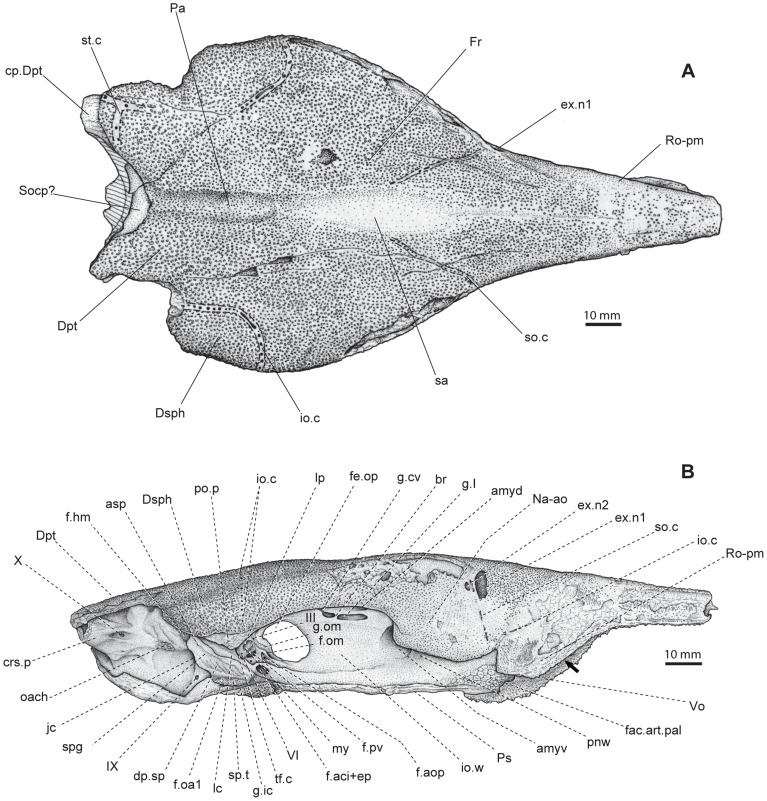
Skull of *Yelangichthys*. Line drawings of GMPKU-P1358 (holotype) in **A**, dorsal; **B**, lateral view. Anterior facing right. **Abbreviations**: **amyd**, anterodorsal myodome; **amyv**, anteroventral myodome; **asp**, ascending process of parasphenoid; **cp.Dpt**, caudal process of Dermopterotic; **crs.p**, craniospinal process; **dp.sp**, depressions in spiracular groove; **Dpt**, dermopterotic; **ex.n1,2**, external naris 1, 2; **f.am**, mandibular adductor muscular fossa; **f.oa1**, foramina of orbital artery; **f.hm**, articular facet for hyomandibular; **f.ic+ep**, foramen of internal carotid artery and efferent pseudobranchial artery; **f.max.buc**, foramen of canal transmitting ramus maxillaris trigemini and ramus buccalis lateralis and vessels; **haem**, notch indicating position of anterior end of haemal canal; **f.om**, foramen of great ophthalmic artery; **f.opa**, foramen of optical artery; **f.pv**, foramen of pituitary vein; **fac.art.pal**, articular facet for autopalatine; **pnw**, postnasal wall; **fe.op**, optical fenestration; **Fr**, frontal; **g.cv**, groove of an unnamed vessel; **g.I,** groove for olfactory nerve; **g.ic**, groove for internal carotid artery; **br**, bony bridge of olfactory groove; **g.om**, groove for great ophthalmical; **io.c**, infraorbital sensory canal and its foramina; **i.ow**, interorbital wall; **jc**, posterior opening of jugular canal; **lc**, lateral commissure; **my**, posterior myodome; **Na-ao**, nasaloantorbital; **oach**, area of origin of dorsal hyoid constrictor muscle; **Pa**, parietal; **po.p**, postorbital process; **Ps**, parasphenoid; **Ro-pm**, rostropremaxilla; **sa**, smooth area on dermal skull roof; **so.c**, openings of supraorbital sensory canal; **Socp**?, supraoccipital?; **sp.t**, spiracular teeth; **spg**, spiracular groove; **stc**, supratemporal commissure of lateral lines; **tf.c**, trigeminofacialis chamber; **tp**, teeth plate on ventral side of parasphenoid; **Vo**, vomer; **II**, optical nerve or its canal; **III**, foramen of oculomotor nerve; **VI**, foramen of abducens nerve; **IX**, foramen of glossopharyngeal nerve; **X**, foramen of vagus nerve.

**Figure 4:**
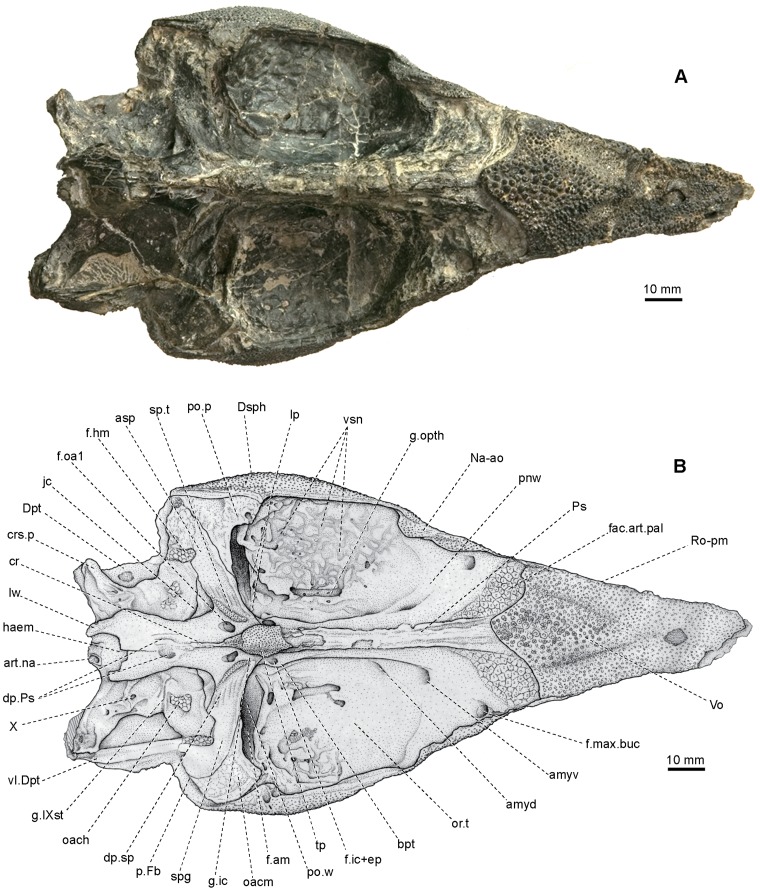
Skull of *Yelangichthys*. Photograph (**A**) and line drawing (**B**) of GMPKU-P1358 (holotype) in ventral view. **Abbreviations**: **art.na**, articular facets of neurocranium with first neural arch; **cr**, crest on ventral side of posterior stem of parasphenoid; **lw**, posterolateral wings of parasphenoid; **g.IXst**, groove for ramus supratemporal lateralis; **g.opth**, groove for ramus ophthalmicus trigemini and ramus ophthalmicus lateralis and some vessels; **oacm**, area of origin of undifferentiated dorsal mandibular constrictor muscle; **or.t**, orbital tetum; **p.Fb**, posterior opening of fossa Bridgei; **pow**, postorbital wall; **vl.Dpt**, ventral lamina of dermopterotic; **vsn**, network of vermiculate sulci on ventral side of orbital tectum; See Fig. 3 for other abbreviations.

**Figure 5:**
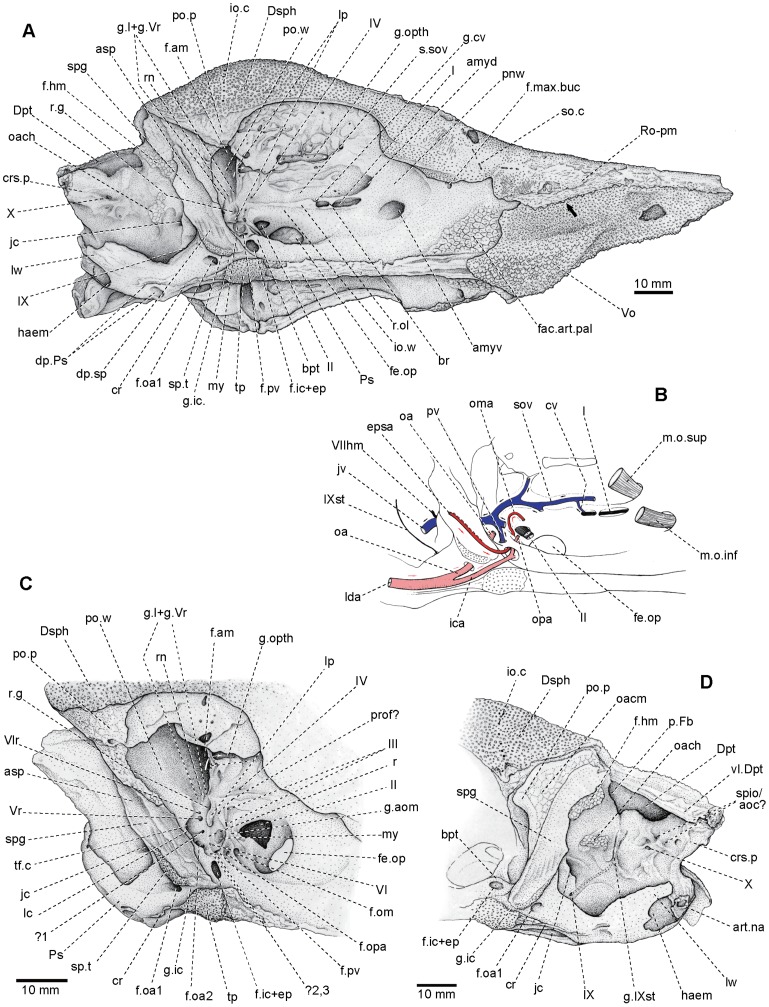
Skull of *Yelangichthys*. Line drawings of GMPKU-P1358 (holotype) in **A**, posterolateral view; **B**, restoration of some nerves and vessels in orbitotemporal region with the palatine branch of internal carotid artery removed, and **C**, posteroventral corner of orbitotemporal region in anteroventral view; **D**, otic and occipital region. **Abbreviations**: **cv**, unnamed vessel; **epsa**, efferent pseudobranchial artery; **g.l+gVr**, grooves for ramus ophthalmicus trigemini and ramus ophthalmicus lateralis and supraorbital artery; **ica**, internal carotid artery; **m.o.inf**, inferior obliqus muscle; **m.o.sup**, superior obliqus muscle; **oa**, orbital artery; **olf.l**, ridge-like bulging behind olfactory groove; **oma**, great ophthalmical artery (arteria ophthalmicus magna); **opa**, optical artery ( = central retinal artery); **prof**, foramen for profundus nerve; **pv**, pituitary vein; **r.g**, recess for trigeminal and lateralis ganglia; **r**, ridge between orbital openings of trigeminofacialis chamber and posterior myodome; **rn**, ridge separating two grooves; **spio/aoc?**, spino-occipital nerve or occipital arteries; **s. sov**, sulcus for supraorbital vein; **I**, olfactory nerve; **IV**, foramen of trochlear nerve; **Vr**, foramen of trigeminal root; **Vlr**, foramen of lateralis root; **VIIhm**, hyomandibular branch of facial nerve; **IXst**, ramus supratemporal lateralis. Anterior facing right in **A**, **B**, **C**, and left in **D**. See Figs. 3 and 4 for other abbreviations.

**Figure 6:**
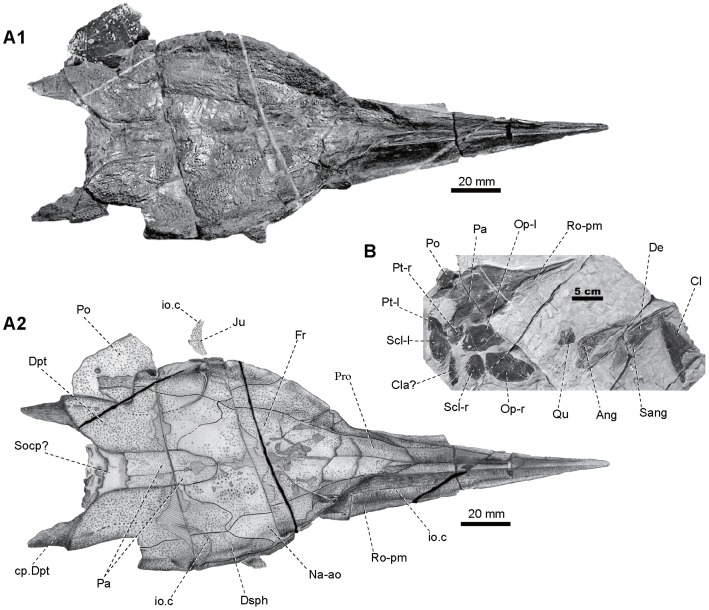
Skull of *Yelangichthys.* Photograph (**A1**) and line drawing (**A2**) of skull (GMPKU-P1359) in dorsal view and photograph (**B**) of GMPKU-P1359. Anterior facing right. **Abbreviations**: **Ang-l**, left angular; **Cla?**, clavicle?; **Cl-r**, right cleithrum; **De-l, -r**, left and right dentary; **Ju**, jugal; **Op-l**, **-r**, left and right opercle; **Po**, preopercle; **Pro**, postrostral; **Pt-l**, **-r**, left and right posttemporal; **Qu-l**, **r**, left and right quadrate; **Sang-l**, left surangular; **Scl-l**, **-r**, left and right supracleithrum. See Figs. 3, 4 and 5 for other abbreviations.

**Figure 7:**
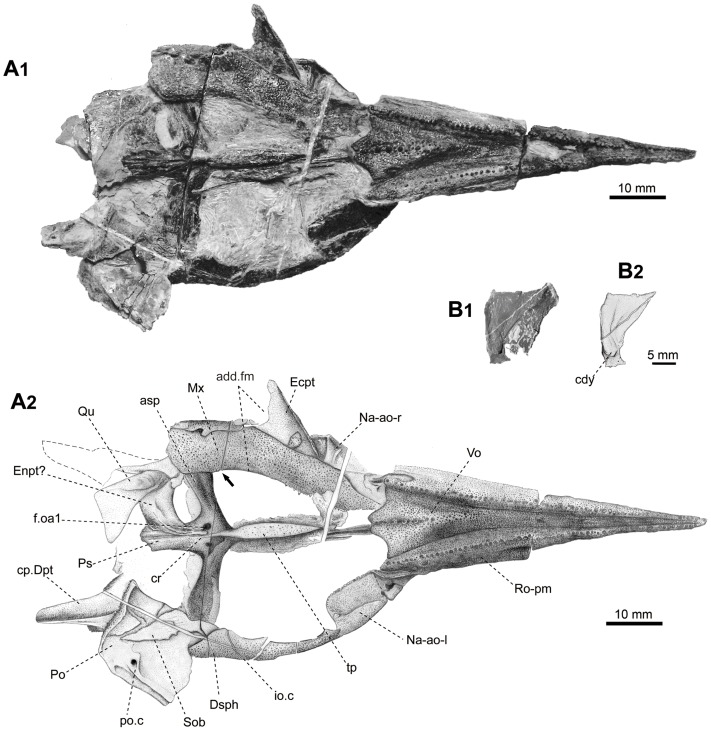
Skull of *Yelangichthys*. Photograph (**A1**) and line drawing (**A2**) of skull (GMPKU-P1359) in ventral view; Photograph (**B1**) and line drawing (**B2**) of left quadrate in medial view. Anterior facing right. **Abbreviations**: **cdy**, condyle of quadrate; **Mx**, maxilla; **Ecpt**, ectopterygoid; **Enpt**, entopterygoid; **Qu**, quadrate. See Figs. 3, 4, 5, and 6 for other abbreviations.

**Figure 8:**
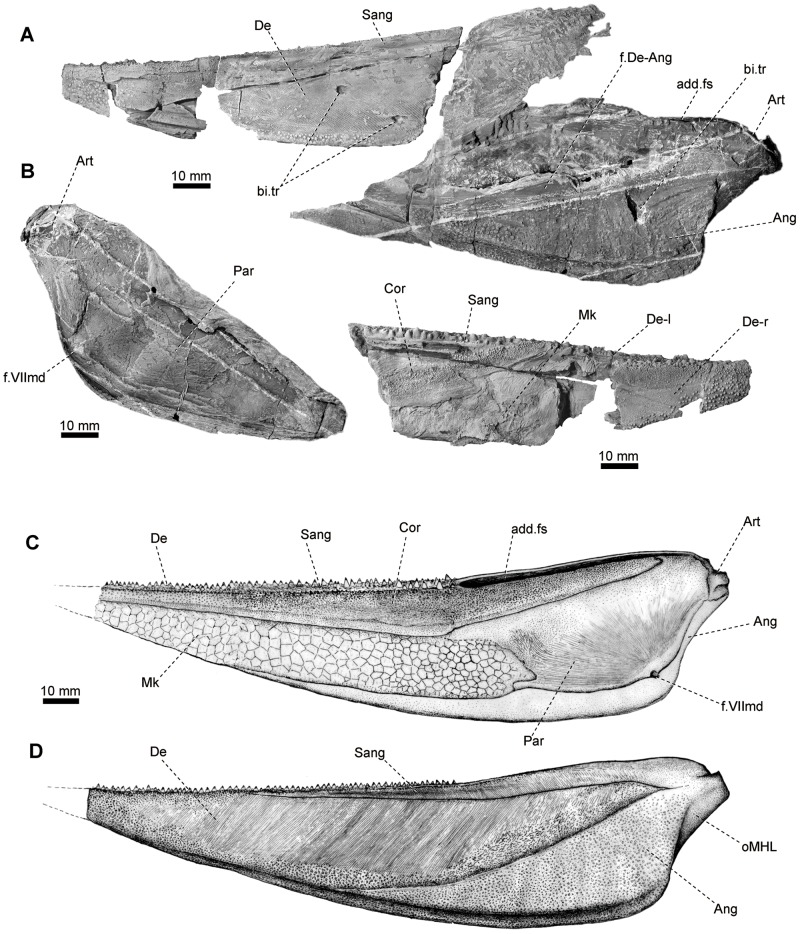
Lower jaw of *Yelangichthys*. Photographs and line drawings of GMPKU-P1359. **A**, mandible elements in lateral view, and **B**, in medial view; **C**, restoration of mandible in medial view, and **D**, in lateral view. Anterior facing right in **B**, and left in rest. **Abbreviations**: **add.fs**, adductor mandibulae fossa; **add.fm**, adductor foramen in upper jaw; **Art**, articular ossification; **bi.tr**, bite trace(s); **Cor**, coronoid; **f.VIImd**, foramen of mandibular branch of facial nerve; **Mk**, remains of Meckelian bone; **oMHL**, attaching area of the mandibulohyoid ligament; **Par**, prearticular.

**Figure 9:**
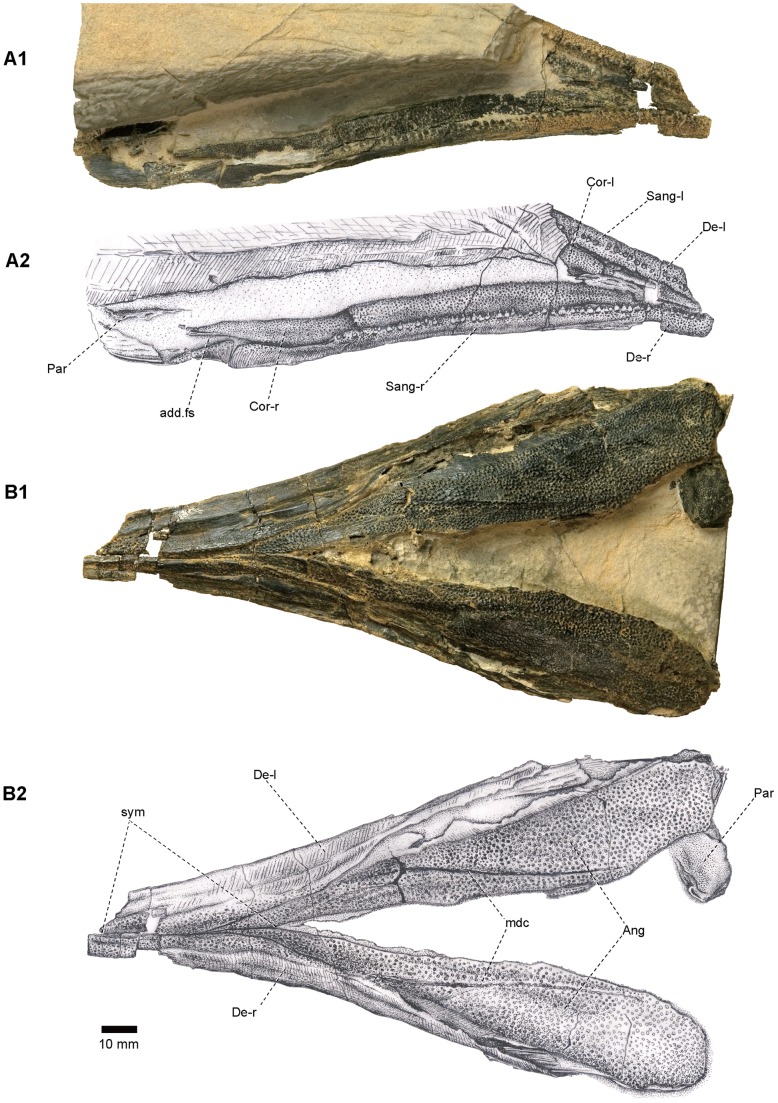
Lower jaw of *Yelangichthys*. Photographs (**A1**, **B1**) and line drawings (**A2**, **B2**) of GMPKU-P1360 in **A**, dorsal, and **B**, ventral view. Anterior facing right in **A**, and left in **B**. **Abbreviations**: **mdc**, mandibular sensory canal; **sym**, mandibular symphysis. See Fig. 8 for other abbreviations.

**Figure 10:**
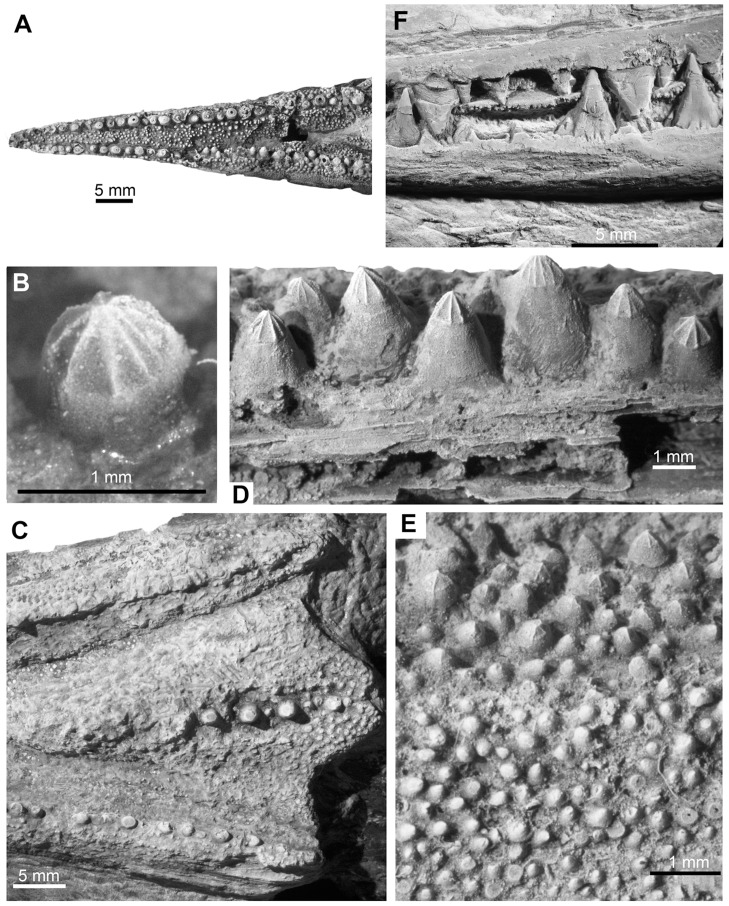
Teeth of *Yelangichthys*. Teeth in **A**, anteriormost rostral portion; **B**, blowup of a tooth of rostropremaxilla in dorsolateral view; **C**, teeth of surangular in lingual view; **D**, posteroventral ethmoidal region in palatal view; **E**, teeth on left coronoid in lingual view; All from GMPKU-P1359 and anterior facing left; **F**, anterior part of jaws of *Saurichthys* sp. from the same locality.

**Figure 11:**
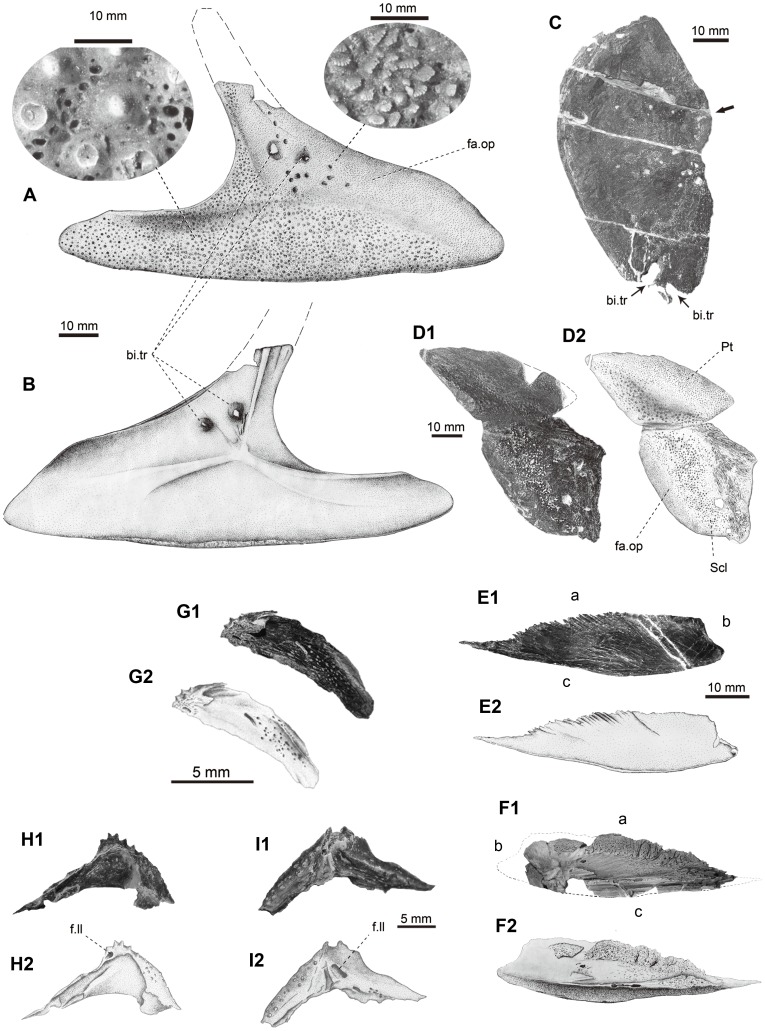
Pectoral girdle and some other bones of *Yelangichthys*. Photographs and line drawings of GMPKU-P1359 (paratype). Right cleithrum in **A**, lateral, and **B**, medial view; **C**, right opercle in lateral view; **D**, left posttemporal and supracleithrum in dorsolateral view; **E**, **F**, clavicle? (**F1**, photograph, and **F2** restoration of **F1**); **G**, **H**, **I**, scales? Anterior facing right in **A**, **C**, left in **B**, **D**, and uncertain in rest. **Abbreviations**: **fa.op**, opercle-covering facet; **f.ll**, foramen of lateral line canal. See Fig. 8 for other abbreviations.

**Type and only known species.** *Yelangichthys macrocephalus* gen. et sp. nov.

#### Diagnosis

A saurichthyiform with massive skull, roof flat and broad behind rostral part; anterior part of braincase low; posterior part of vomer bending down abruptly together with deepened braincase; orbitotemporal region distinctly longer and broader than oticoccipital region; orbital tectum broad, almost reaching lateral margin of dermal skull roof; peculiarly deep and narrow, transverse fossa in posterodorsal part of orbit; before extending anteriorly into the canal towards nasal cavity, olfactory nerve going in deep groove in interorbital wall after emerging from cranial cavity; ramus ophthalmicus trigemini and ramus ophthalmicus lateralis separated in orbital region; posterior myodome paired; both mandibular adductor foramen in upper jaw and adductor fossa in lower jaw long, extending anteriorly to level of posterior part of orbit; surangular very long, forming part of tooth-bearing mouth margin in front of adductor fossa; angular partaking in mandibular symphysis; coronoid partaking in adductor fossa; dentary not partaking in mandibular adductor fossa; all teeth caps with screwdriver-like tips.

#### Etymology

*Yelang*, Pinyin spelling of the name of an ancient kingdom in southern Guizhou, and Greek *ichthys*, meaning fish; *macrocephalus*, referring to its large head.

#### Holotype

GMPKU-P1358, a three-dimensionally preserved braincase with dermal skull roof *in situ*, but the anterior part of the rostrum missing.

#### Paratype

GMPKU-P1359, a flattened skull with disarticulated elements of lower jaw and dermal pectoral girdle.

#### Type locality and horizon

Xindian, Qingshan, Pu'an County, Guizhou Province,

China; CY12 of the Upper Member of the Guanling Formation (Fig. 1).

### 2. Description and comparison

#### 2.1 General appearance of the braincase

As in saurichthyids, the skull has a very long snout, tapering to the anterior extremity. The dermal bones are ornamented mainly with similar tubercles and striations as those in saurichthyids. The neurocranium is composed of one large single ossification without any distinct sutures or fissures, of which the orbitotemporal region occupies a fairly large proportion, whereas the oticoccipital region is rather short (Figs. 2–5). This peculiar configuration makes the skull look distinctly different from those of known saurichthyids and other actinopterygians.

The braincase of the holotype (GMPKU-P1358), with the anteriormost rostral portion broken, is measured about 180 mm long and 100 mm wide, whereas the maximal depth at the posteriormost ethmoidal region, where the vomer bulges downward, is 35 mm. The skull roof of the paratype (GMPKU-P1359, Fig. 6A, 6B) is completely preserved but smaller than that of the holotype, with the maximal length as 220 mm and width as 89 mm. The specimen GMPKU-P1360 is paired articulated mandibular rami with the anteriormost part lost. A comparison of the size of the dermal mandibular bones between the latter two specimens indicates that they most probably belong to individuals of similar size and smaller than that of the holotype.

#### 2.2 Skull roof

The dermal skull roof is flat and broad, consisting of the frontals, parietals, and the dorsal part of the nasalo-antorbitals and dermopterotics. The sutures between these bones are clear in GMPKU-P1359 (Fig. 6A_1_, A_2_) but obscure in P1358 (Figs. 2A, 3A). The skull roof is ornamented with coarse tubercles, except for an elongate depressed smooth area (sa, Fig.3A) in the medial part of the frontals.

The paired frontals (Fr, Fig. 6A_2_) nearly occupy the anterior half of the skull roof with its narrow anterior portion extending forward along the dorsal edge of the nasalo-antorbitals to meet the postrostrals (Pro, Fig. 6A_2_). The frontal increases gradually in width posteriorly and meets the dermopterotic in a sinuous line. The parietals (Pa, Fig. 6A_2_) wedge in between the frontals from behind. The depressed smooth area (sa, Figs. 2A, 3A) extends along the midline from the level of posterior border of the optical fenestra to that of the anterodorsal myodome of the neurocranium.

In GMPKU-P1359, the parietals (Pa, Fig. 6A_2_) occupy the posterior medial portion of the skull roof, with the left one slightly larger than the right. They have a round anterior end and suture with the dermopterotics laterally and frontals anteriorly. In P1358, the parietal region is somewhat depressed and covered with dense but relatively small tubercles. No extrascapular is preserved and part of the posteriormost occipital region of the neurocranium is exposed (Socp?, Figs. 3A, 6A_2_).

The dermopterotics (Dpt, Figs. 3A, 6A_2_) constitute the posterolateral portion of the skull roof. They do not meet with each other behind the parietals, differing from those in the majority of saurichthyids but similar to those in *Saurichthys piveteaui* from Madagascar [17], *S. obrutchevi* and *S. proximus* from Russia [38], and *S. orientalis* from Central Asia [39]. The dermopterotic bears a distinct notch in its lateral margin and a triangular, posteriorly-directed process behind the lateral caudal corner (cp.Dpt, Figs. 3A, 6A_2_, 7A) comparable to the facet receiving the dorsalmost element of the pectoral girdle in saurichthyids [3]. On the ventral side of this corner is a massive strut (vl.Dpt, Figs. 4B, 5D) extending posteroventrally until it meets the distal end of the craniospinal process (crs.p, Figs. 4B, 5D) of the neurocranium.

Different from those in saurichthyids, the nasalo-antorbital (Na-ao, Fig. 6A_2_) and dermosphenotic (Dsph, Fig. 6A_2_) both have a large dorsal part that partakes in the dermal skull roof, thereby making a much broader skull table in proportion.

#### 2.3 Snout

In GMPKU-P1359 the snout is well-preserved (Figs. 6, 7). It is very elongated and covered externally by the rostro-premaxillae, nasalo-antorbitals, and paired postrostrals.

The rostro-premaxilla (Ro-pm, Figs. 6A_2_, 7A) is triangular, tapering forward to the anterior tip, with the infraorbital sensory canal (io.c, Figs. 3B, 6A_2_) traversing throughout, the anteriormost part of which cannot be seen clearly. The bone meets the postrostral (Pro, Fig. 6A_2_) posteromedially and nasalo-antorbital (Na-ao, Figs. 3B, 6A_2_) posterolaterally, and is fused with its antimere in the anterior third length. Externally, it is furnished mainly with anteriorly-inclined striations and some tubercles along its dorsal edge and in the anterior extremity. It is very interesting that the oral edge of this bone is mostly straight except where the ventral protrusion in the posterior ethmoid region occurs (arrowed in Figs. 3B, 5A).

The nasalo-antorbital (Na-ao, Figs. 3B, 6A_2_) consists of a dorsal and a lateral portion, forming part of the skull roof dorsally and contacting the dermosphenotic posteriorly. Two subovate external nares open within its lateral portion (ex.n1, 2, Fig. 3B) with the anterior one larger than the posterior one as in other saurichthyiforms. The rhombic lateral portion is ornamented mainly with fine striations and a few tubercles along its edges and the major part of the antorbital region, with its posterior edge forming the anterior rim of the orbit.

The paired postrostrals (Pro, Fig. 6A_2_) suture with the frontals posteriorly, and with the nasalo-antorbitals laterally, thus separating the frontals from the rostro-premaxillae, a condition similar to that in *Saurorhynchus acutus* [18]. The numbers of the postrostrals are variable among saurichthyiforms. They are paired in *Saurichthys* species from Spitzbergen [1] and in *Saurorhynchus acutus* [18] and numerous in *S. stensiöi* and *S. piveteaui* [17], [40], but absent or not recognizable in most of other species [2], [3], [22], [41].

#### 2.4 Dermal bones on ventral side of the neurocranium

The parasphenoid and the single median vomer are almost completely preserved in both GMPKU-P1358 and P-1359. The parasphenoid (Ps, Figs. 3B, 4B, 5A, 7A_1_, A_2_) is of a general topology similar to that of *Saurichthys* [1], consisting of an elongated anterior stem attached to the ventral side of the orbitotemporal region, a pair of ascending processes (asp, Figs. 3B, 4B, 7A_2_) stretching posterodorsolaterally along the lateral side of the lateral commissure (lc, Figs. 3B, 5C), and a posterior stem extending posteriorly under the otic and occipital region of the neurocranium. The anterior stem occupies about 60% of the total length of the parasphenoid corpus, bearing on the ventral side a lanceolate tooth plate (tp, Figs. 4B, 5A, 5C, 7A_2_) and a low median ridge with paired flanges close behind the vomer (Vo, Figs. 4B, 5A, 7A_2_). The bucco-hypophysial opening is not observed. The ascending process (asp, Figs. 3B, 4B, 5A, 5C, 7A_2_) is constructed with the spiracular groove (spg, Figs. 3B, 4B, 5A, 5C, 5D) running along its outer face. Between its base and the parasphenoid corpus is the groove (g.ic, Figs. 3B, 4B, 5A, 5C, 5D) for the internal carotid artery (ica, Fig. 5B), above which is a tooth patch (sp.t, Figs. 3B, 4B, 5A, 5C) delimiting the posterior boundary of the spiracular groove. More dorsally in the spiracular groove, at the level of the trigeminofacialis chamber (tf.c, Figs. 3B, 5C), there are five to six narrow depressions (dp.sp, Figs. 3B, 4B, 5A) alternating with low ridges arranged in a direction diagonal to the axis of the ascending process. The posterior stem of the parasphenoid, slightly less than one-third of the corpus length, possesses a median ventral crista (cr, Figs. 4B, 5A, 5C, 5D, 7A_2_) and a pair of caudal wings (lw, Figs. 4B, 5A, 5D), which are preserved in such a way in the holotype that they are curved upward to wrap the hindmost part of the occipital region. The crista referred above must have separated the paired lateral dorsal aortae, similar to that in *Sinosaurichthys* [3] from the same fauna, and other saurichthyids [1], [18]. Proximally the caudal wings form a round notch as the anterior end of the haemal canal (haem, Figs. 4B, 5A, 5D) as in saurichthyids [1]. Nearly in the midway of the posterior stem, there is a pair of subovate depressions (dp.Ps, Figs. 4B, 5A) behind the ventral crista, though the right one of them is not well preserved, of which the function is not known. Posteroventral to the ascending process is the lower opening (f.oa1, Figs. 3B, 4B, 5C, 5D) of the canal, which extends anterodorsomedially to open in the ventral part of the trigeminofacialis chamber (f. oa2, Fig. 5C). This is a similar topology seen in some saurichthyids; however, the assignment for this canal is very different: either as for containing the common carotid arteries [15], [36], or for the ‘external carotid arteries’ [1], [12], [18], [37] (but using of this name should be avoided because the artery herein may be not the external carotid artery but the orbital artery, for whose definitions see [42]). Here we adopt Stensiö's idea (though not his terminology), i.e., the lateral dorsal aorta ( = arteria carotis communis in Stensiö's nomenclature [1]) (lda, Fig. 5B) gives off the orbital artery (oa, Fig. 5B), which extends in the canal towards and then branches within the trigeminofacialis chamber (tf.c, Figs. 3B, 5C) as it is usual in actinopterygians [37], [43], whereas the remaining part, the internal carotid artery (ica, Fig. 5B), continues forward ventrally to the parasphenoid rather than dorsally as in the majority of other actinopterygians. Then the latter artery enters the cranial base together with the efferent pseudobranchial artery (epsa, Fig. 5B), a character shared by saurichthyids, *Acipenser*, *Polyodon* [1], [44] and *Birgeria* [45].

The vomer (Vo, Figs. 3B, 4B, 5A, 7A_2_) is roughly of a triangular shape, tapering anteriorly and widening posteriorly. Its lateral margins are flanked by the tooth-bearing edge of the rostro-premaxillae (Ro-pm, Figs. 3B, 4B, 5A, 7A_2_) and its posterior border looks like the letter ‘W’ with the median tine pointing anteriorly, as the place meeting the parasphenoid. On the oral surface of the vomer, numerous small teeth and a median row of larger ones are seen in GMPKU-P1359 (Fig. 7A), whereas in GMPKU-P1358 the larger teeth cluster together in the posteromedian part (Figs. 4B, 5A). Remarkably, the posterior part of the vomer in this specimen bends down so much that it looks like a protuberance in the lateral view (Figs. 2B, 3B, 5A). The median structure and its relation with the parasphenoid are different from those in saurichthyids whose parasphenoid wedges between the paired vomers for a considerable distance [1], [3], [18].

#### 2.5 Ethmoid region of neurocranium

Most anterior part of the ethmoid region is covered by the external dermal bones, and only the postnasal wall and the posterior part of the ventral surface are exposed. The postnasal wall (pnw, Figs. 3B, 4B, 5A) is gently concave and equally divided by the anterior end of the interorbital wall (io.w, Figs. 3B, 5A). Close to the transition between these two walls are two pairs of anterior myodomes (amyd, amyv, Figs. 3B, 4B, 5A), housing the origins of the superior and inferior oblique eye muscles, respectively (m.o.sup, m.o.inf, Fig. 5B). The dorsal myodome is situated posteromedial to the ventral and with a broad and shallow sulcus following it (s.sov, Fig. 5A). The interorbital wall (io.w, Figs. 3B, 5A) in this area is so thick that the myodomes seem to be separated from their antimeres. There is no opening for the olfactory canal in the postnasal wall as in saurichthyids [1]. Without any distinct transition, the exposed ventral surface of the ethmoid region extends posterodorsally to be confluent with the postnasal wall, with a rough, triangular depression on each side as the articular facet for the autopalatine (fac.art.pal, Figs. 3B, 4B, 5A). Just posterolateral to this facet, there is a foramen (f.max.buc, Figs. 4B, 5A), comparable to the posterior opening of the canal lodging the ramus maxillaris trigemini, ramus buccalis lateralis and some vessels in *Saurichthys* [1], *Pteronisculus* ( =  *Glaucolepis* [40]) and *Boreosomus* [46], which extends forward accompanying the infraorbital sensory canal in the snout.

The most striking in the ethmoid region is the structural abnormity in the posterior part. Distinctly different from the low anterior portion of braincase, the braincase deepens abruptly at the level of the posterior part of vomer, which also bends down at this position (Figs. 2B, 3B, 5A), corresponding to the downward curve of the rostro-premaxilla referred above. This unique configuration of the mouth of *Yelangichthys* is to us a structural innovation related with its durophagous dietary habit and this area should be a structure to crack and crush the prey items functioning like a pestle (see discussion below).

#### 2.6 Orbitotemporal region of neurocranium

On account of the considerable size of the orbits, the orbitotemporal region in *Yelangichthys* is quite large, occupying about one-third of the total length of the neurocranium. The region is bounded anteriorly by the postnasal wall, and posteriorly by the steep postorbital wall (po.w, Figs. 4B, 5A, 5C). This region is peculiar in having a very broad orbital tectum (or.t, Fig. 4B), which extends nearly as laterally as the dermal skull roof does, and a unique large and deep transverse fossa in the posterodorsal part of the orbit (f.am, Figs. 4B, 5A, 5C).

Different from that in saurichthyids, the interorbital wall (io.w, Figs. 3B, 5A) is rather thick, especially in its anterior part. It thickens upwards until it merges into the orbital tectum laterally and the postnasal wall anteriorly. The optic fenestra (fe.op, Figs. 3B, 5A, 5C) penetrates the interorbital wall in the posteroventral part of the orbit, situated more posteriorly than in saurichthyids [1]. The fenestra is bordered posteriorly by the narrow *pars basisphenoidea* (the region between the optic fenestra and posterior myodome (my, Figs. 3B, 5A, 5C)). It is somewhat elliptical with its long axis nearly horizontal, and measures about 15 mm long and 10 mm high, which is rather small as compared to the orbital size. Neither emargination nor separate opening emerges at the anterodorsal edge of the fenestra, though one or the other case is often seen in many saurichthyids [1]; however, a distinct notch occurs in the posterodorsal margin, indicating the exit of the canal for the optic nerve (II, Figs. 3B, 5A–C).

The orbital tectum (or.t, Fig. 4B) is the widest approximately at the level of the center of the optic fenestra, with a convex lateral margin. It gradually narrows anteriorly and posteriorly till it merges into the dorsolateral edge of the postnasal wall and the anterodorsal portion of the postorbital process (po.p, Figs. 3B, 4B, 5A, 5C, 5D). Its large width is reminiscent of that in some primitive osteichthyans *Ligulalepis*, *Guiyu*, *Powichthys*, *Youngolepis*, and *Psarolepis* [47]–[52], but is distinct from that in the majority of other known actinopterygians and sarcopterygians [1], [12], [17], [36], [37], [46], [47], [53]–[55], whose orbital tectum is always constricted in the interorbital portion and is thus rather narrow or even not developed at all. On the underside of the orbital tectum is a network of numerous anastomosing grooves and foramina of various sizes, from which we can trace the pathways and the arrangement of some nerves and vessels described below (g.opth, vsn, Figs. 4B, 5A).

The large transverse fossa in the posterodorsal part of the orbit (f.am, Figs. 4B, 5A, 5C) is about 22 mm wide and five mm long and cutting deeply into the orbital tectum from below just in front of the postorbital wall. Its anterior and posterior walls are lined with periosteal bone, whereas its dorsal part is rough. This fossa is so deep that it almost reaches the skull roof. This is a very special structure among the well-known actinopterygians or even osteichthyians and its function will be discussed below. Although a recess in the similar area, which was not labeled in the figures or even not mentioned in the literature, is also observable in the Devonian *Mimipiscis* ( = *Mimia*)[12], [56], Permian *Luederia* [57] and Triassic *Pteronisculus* [58], it is much less notable than the fossa in *Yelangichthys* and should not be homologous to the fossa herein.

The ventral edge of the anterior wall of the fossa mentioned above is a narrow but pronounced ridge, which begins from a small process (lp, Figs. 4B, 5A, 5C) hanging above a low crest (r, Fig. 5C) between the posterior myodome and trigeminofacialis chamber, and extends anterodorsolaterally first and then posterodorsolaterally until it merges into the base of the postorbital process (po.p, Figs. 4B, 5A). This ridge most likely represents the vestigial lateral pillar (lp, Figs. 4B, 5A, 5C), which is the derivation from the suprapharyngomandibular according to Jarvik [37] based on the embryological study of *Amia*, with the ramus ophthalmicus superfacialis trigeminus and lateralis perforating its upper part. Certain vestige of the pillar, named as alisphenoid (or pterosphenoid) pedicle, or the lateral pillar itself can be also seen in *Myothomasia* [12], *Kansasiella* [37], [43], [59], *Birgeria* [53], and *Sinamia* [60]. By contrast, comparable pillar or pedicle does not exist in *Polypterus* [54], [61], *Acipenser* [61], *Lepisosteus* [61], [62] or *Pteronisculus* [58], [46].

In the holotype, the right side of the orbitotemporal region is better preserved and more properly prepared than the left, though the posterior part of the orbital tectum is somewhat deformed.

The olfactory canal opens anterodorsal to the optic fenestra, leading from which is a deep groove (g. I, Figs. 3B, 5A, 5B) straddled by a thin bony bridge (br, Figs. 3B, 5A). Anteriorly, this groove enters a relatively large foramen posteroventral to the anterodorsal myodome. And behind the groove is a pronounced ridge tapering off posteriorly (r.ol, Fig. 5A). From the posterodorsal side of the olfactory groove is a tiny groove (g.cv, cv, Figs. 3B, 5A, 5B) extending towards a sulcus close above (s.sov, Fig. 5A), probably indicating the course of an unknown vessel from the cranial cavity. The course of the olfactory nerve in the orbital region is different in saurichthyids: it exits from the postnasal wall and traverses a long distance in the orbit before it enters into the cranial cavity, leaving no traces on the interorbital wall [1]. Otherwise, it usually extends in other fishes within the bone-enclosed canals in the interorbital wall [12], [36], [37], [43], [47], [50]–[55], [63]–[69].

The anterior end of the cranial cavity must be situated somewhere behind the olfactory foramen anterodorsal to the optic fenestra (fe.op, Fig. 5A), and the cavity starts to enlarge roughly at the boundary between the orbitotemporal and otic regions at the level of the mid-brain. However, no more detailed information was obtained about the structure of the cavity except the absence of the buccohypophysial foramen on the underside of the neurocranium, which is present in saurichthyids, but not in *Acipenser* and *Polyodon* [1].

Similar to those in *Saurichthys wimani*, *S. hamiltoni*, *S. elongatus*, and many other actinopterygians, the optic canals (II, Fig. 5A, 5C) of both sides exit from the braincase through a single, wide opening above the *pars basisphenoidea*, without a tongue-shaped bone as seen in some *Saurichthys* species (e.g., *S. ornatus*) which divides the opening into two foramina. The external opening of the optic canal is the posterodorsal notch of the optic fenestra (fe.op, Figs. 3B, 5A, 5C).

Two small foramina of the oculomotor nerve (III, Figs. 3B, 5C) are located posterior to the external opening of the optic canal in the way that one is above the other. Slightly anterodorsal to these foramina is the exit of the trochlear nerve (IV, Fig. 5A, 5C).

On the posteroventral side of the orbitotemporal region, just under the *pars basisphenoidea* and lateral to the parasphenoid is an ear-like endoskeletal basipterygoid process (bpt, Figs. 4B, 5A, 5D) penetrated by a large foramen (f.ic+ep, Figs. 3B, 4B, 5A, 5C, 5D), which must have received both the internal carotid artery (ica, Fig. 5B) and the efferent pseudobranchial artery (epsa, Fig. 4B). After entering the foramen, the former goes upwards into the cranial cavity, whereas the latter extends first upward and then forward to the orbit through the foramen (f.om, Figs. 3B, 5C) as the ophthalmic magna artery (aom, Fig. 5B), from which foramen emerges a curving groove (g.om, Figs. 3B, 5C) extending in the way shown in the figures until it tapers off above the opening of the optic canal. Slightly lateral to the foramen for the ophthalmic magna artery is a smaller foramen (f.opa, Figs. 3B, 5C), likely for the optical artery (opa, Fig. 5C).

The trigeminofacialis chamber (tf.c, Figs. 3B, 5C) and the posterior myodome (my, Figs. 3B, 5A, 5C) are in the posteroventrolateral part of the orbitotemporal region. The chamber lies between the lateral cranial wall and the lateral commissure (lc, Figs. 3B, 5C). The chamber is separated from the myodome by a low ridge (r, Fig. 5C), which is extending downwards from the lower end of the vestigial lateral pillar (lp, Fig. 5A, 5C). The posterior myodome is separated from its antimere, with a low oblique ridge in it and the exit of the abducens nerve (VI, Figs. 3B, 5C) posteroventral to it. In this case, the posterior myodomes are paired, in contrast to the single one in saurichthyids [1], [17], and the insertion of the external rectus muscle did not obliterate the pituitary canal. Under the myodome is the opening of the pituitary canal (f.pv, Figs. 3B, 5A, 5C and pv, Fig. 5B). However, whether some parts of the external rectus muscle had entered this canal cannot yet be confirmed. Just below the opening of the pituitary canal are two undetermined foramina (?2, 3, Fig. 5C).

The good exposure of the anterior part of the trigeminofacialis chamber makes the following structural observations possible. Two foramina, the dorsal one being much larger than the ventral, are clearly shown in the anterior part of the medial wall of the chamber, just behind the lower part of the vestigial lateral pillar (lp, Fig. 5C). These two foramina are comparable to those in *Saurichthys ornatus*, which transmit the trigeminal root proper (the general cutaneous and motor V fibers) and several branches from the preauditory lateral nerve root (ramus ophthalmicus lateralis and ramus buccalis lateralis), respectively (Vr, Vlr, Fig. 5C). More ventrally is a small undetermined foramen with a groove from behind (?1, Fig. 5C), and just below the foramen is the exit of the orbital artery (oa, f.oa2, Fig. 5B, 5C).

A rounded recess is between the two foramina referred above (Vr, Vlr, Fig. 5C) and the lower end of the lateral pillar (lp, Fig. 5C), which presumably indicates the position of the trigeminal and lateral ganglia (r.g, Fig. 5A, 5C). From this recess, two grooves (g.l+g.Vr, Fig. 5C), partially divided by a low ridge (rn, Fig. 5A, 5C), extend dorsally. These two grooves must have lodged the ramus ophthalmicus superfacialis trigemini and ramus ophthalmicus lateralis, and some related vessels, and should be eventually associated with the longitudinal groove (g.opth, Figs. 4B, 5A, 5C) in the underside of the orbital tectum (arrowed in Fig. 5A, 5C), a judgment based on the following observations: lateral to the groove, the ventral side of the orbital tectum bears a network of ramified and anastomosing grooves of different calibers, with a few of them connected with the large groove (g.opth, Figs. 4B, 5A). Small pores are seen in both the large and the ramified smaller grooves, and the large groove is roughly under the supraorbital canal in the dermal skull roof. Such arrangement of these structures matches the usual peripheral distribution of two nerves, the ramus ophthalmicus lateralis and ramus ophthalmicus superfacialis trigemini [1], [37], of which the former innervates the supraorbital canal, whereas the latter, which consists exclusively of cutaneous fibers, is always extensively ramified and extends to the cranial roof. So, if it is true, the large groove referred above must have lodged both of these two nerves from their exits (Vr, Vlr, Fig. 5C) in the trigeminofacialis chamber [1], [37]. The two nerves in question thus ran closely together as usually the case in fishes [1], [12], [21], [37], [53], [64], but different from those in *Saurichthys*, where they are distinctly separated from each other throughout their courses [1].

The exit of the facial nerve cannot be observed because it must have penetrated the medial wall of the posterior part of the trigeminofacialis chamber, and thus is hid by the lateral wall of the chamber, and the state of the geniculate ganglion and the origin of the ramus palatinus facialis cannot be discerned either.

#### 2.7 Otic region of neurocranium

The short otic region, defined between the postorbital wall and the vagus foramen according to Jarvik [37], is widest at the level of the postorbital process and rapidly decreases in width posteriorly till reaching a distinct constriction in the posterior part.

The massive postorbital process (po.p, Figs. 3B, 4B, 5A, 5C, 5D) protrudes posterodorsolaterally to the orbit and anterodorsolaterally to the ascending process of the parasphenoid (asp, Figs. 3B, 4B, 5A, 5C). It has a slightly depressed and posteroventrally-directed facet in the posterior surface, and the facet is continuous without any trace of division, possibly for the undifferentiated dorsal mandibular constrictor muscle to attach (oacm, Figs. 4B, 5D), a similar situation to that in ‘palaeoniscoids’ and living chondrosteans [12]. No dilatator fossa is developed.

Just posterior to the dorsal part of the ascending process of the parasphenoid (asp, Figs. 3B, 4B, 5A, 5C) is the articular facet for the hyomandibular (f.hm, Figs. 3B, 4B, 5A, 5D), which is an oblique, elongated depressed area without perichondral lining. And posterodorsal to this facet, the otic region narrows abruptly to form a deep embayment in the lateral margin, where the fossa Bridgei (p.Fb, Figs. 4B, 5D) seems to open posteriorly. Posteroventral to this facet is the opening of the jugular canal (jc, Figs. 3B, 4B, 5A, 5C, 5D), which originally contains the jugular vein (jv, Fig. 5B) and some nerves, e.g., the hyomandibular branch of the facial nerve (VIIhm, Fig. 5B). Under this opening, between the ascending process and the posterior stem of the parasphenoid there is a lamella, of which the ventral surface is somewhat concave and uneven with a few foramina. Slightly ventral to the hyomandibular facet is an irregular scar-like surface, probably referring to the area of the origin of the undifferentiated dorsal hyoid constrictor muscle (oach, Figs. 3B, 4B, 5A, 5D). No subtemporal fossa is developed. More ventrally, the glossopharyngeal foramen (IX, Figs. 3B, 5A, 5D) is situated closely posteroventral to the opening of the jugular canal (jc, Figs. 3B, 4B, 5A, 5C, 5D), as it is in *Saurichthys* [1]. And from this foramen is a groove (g.IXst, Figs. 4B, 5D) extending posterodorsally, which is comparable to the furrow for the dorsal ramus of the glossopharyngeal lateralis (IXst, Fig. 5B) in *Saurichthys ornatus* [1], i.e., the supratemporal branch of the glossopharyngeal nerve in other lower actinopterygians, such as *Boreosomus* [46], *Kansasiella* [43], *Lawrenciella* [21], a nervous branch innervating the most anterior part of the cephalic division of the lateral line and the middle pit line [1], [37]. The openings of the spiracular canal cannot be distinguished.

#### 2.8 Occipital region of neurocranium

Contrary to the long occipital region in *Saurichthys* [1], the region is rather short in *Yelangichthys*, less than one-tenth of the total length of the braincase. It has a pair of massive, dorsolaterally extending craniospinal processes (crs.p, Figs. 3B, 4B, 5A, 5D), which contacts distally with a ventral lamina (vl.Dpt, Figs. 4B, 5D) on the underside of the dermopterotic on each side. Approximately at the base of this process is a relatively large subovate recess lodging the vagus foramen (X, Figs. 3B, 4B, 5A, 5D).

In the posterior end of the occipital region, two symmetrically arranged facets, devoid of periosteal lining and facing posteroventrally (art.na, Figs. 4B, 5D), most likely represent the articular facets in the rear of the neurocranium, which originally articulates with the first neural arch.

The craniospinal process occupies a considerable width of the posteriormost part of the occipital region. Although it is not totally exposed in the hindmost side in current material, the contour of the braincase in this portion suggests that the posttemporal fossa for the insertion of the trunk musculature must have developed in the similar way as in *Saurichthys* [1] and sturgeons [62], i.e., being floored ventrally and blocked anteriorly by the craniospinal process.

A posterolaterally projecting process is seen near the proximal end of craniospinal process and there are two small foramina situated in the small recesses ventromedial to the base of the craniospinal process, possibly for some spino-occipital nerves or occipital arteries (spio/aoc?, Fig. 5D).

#### 2.9 Cheek bones and opercular apparatus

The dermosphenotic (Dsph, Figs. 3, 4B, 5A, 5C, 5D; 6A_2_) is very large, comprising a lateral and a dorsal portion. The lateral portion overhangs the orbit and meets the nasalo-antorbital anteriorly, whereas the roughly trapezoid dorsal portion partakes in the skull roof. In saurichthyids, this bone is generally small and restricted to the posterodorsal corner of the orbit, and is separated from the nasalo-antorbital either by the supraorbitals in some Lower Triassic and Jurassic forms [1], [19], [70], or by the frontal, where the supraorbitals are absent, e.g., in late Anisian and Ladinian *Saurichthys curionii* [2]. The dermosphenotic bears a series of openings of the infraorbital sensory canal (io.c, Figs. 3, 5A, 5D, 6A_2_) and is decorated with coarse, round tubercles.

A disarticulated jugal is recognized in GMPKU-P1359 (Ju, Fig. 6A_2_) (it was split away from the rock during acid preparation, thus it is absent in Fig. 6A_1_). This roughly triangular bone has a concaved anterior edge and several pores of the infraorbital sensory canal in its ventral and posterior edges (io.c, Fig. 6A_2_).

Bony elements in the postorbital region are only preserved in GMPKU-P1359, including the left preopercle (Po, Figs. 6A_2_, 6B, 7A_2_), part of the right maxilla (Mx, Fig. 7A_2_), and possibly some remains of the suborbital (Sob, Fig. 7A_2_).

The left preopercle (Po, Figs. 6A_2_, 6B, 7A_2_) is preserved partially beneath the dermopterotic (Dpt, Fig. 6A_2_). Its ventral portion is broken so that its suture with the maxilla is not seen. The remaining part is roughly quadrate with slightly concave anterior and nearly straight posterior margins, and a medially bent and relatively short dorsal flange. On the inner side, a roughly vertical ridge close to the posterior margin extends upward and widens into a triangular plate where a small foramen emerges, thereby marking the preopercular canal (po.c, Fig.7A_2_). The lateral surface of the preopercle is ornamented with tubercles slightly smaller than those on the skull roof.

The right maxilla (Mx, Fig. 7A_2_) is slightly concave along its dorsal edge in the middle portion. From the cross section of a fracture on this bone (arrowed in Fig. 7A2), it can be observed that the postorbital portion is folded due to compaction, thus, it is clearly that the maxilla is of a typical ‘palaeoniscoid-like’ profile. On the mouth margin, the main teeth row terminates roughly at the level where the maxilla bends slightly upwards. When compared with saurichthyids, this configuration of the maxilla correspond to an anteriorly extended adductor foramen (add.fm, Fig. 7A_2_) in the upper jaw [1], [3], and the foramen is delimited laterally by the maxilla and anteriorly by the rounded, concaved posterior margin of the ectopterygoid (Ecpt, Fig. 7A_2_). This foramen is thus fairly long, a feature corresponding to the prolonged mandibular adductor fossa in the lower jaw and the large fossa in the posterodorsal part of the orbit (see below). In the anteriormost part of the maxilla, an area originally overlapped by the rostro-premaxilla is exposed, with the suture inclined posteriorly. The external surface of the maxilla bears many tubercles and some nearly vertical fine striations in the posterodorsal portion.

The opercle (Op-l, -r, Figs. 6B, 11C) is roughly oval with a gently round posterior, a straight anterodorsal, and a slightly convex anteroventral margin. No independent subopercle is developed as in saurichthyids [1]–[4], [18], [19], [32], [38], [40], [41], [70], [71]. The existence of the antopercle remains to be seen. The ornament of the opercle consists mainly of numerous round pits, and a few irregular, anastomosing striae along the margins.

#### 2.10 Mandible

The mandible is massive and of a similar profile with that of saurichthyids, but is unique in having exceptionally long surangular, adductor fossa, and mandibular symphysis involving the angulars. Since the skull roof is much broader than the rostrum, the mandibles are slightly bent laterally at the level where the skull broadens, similar to the shape of the mandibles of the long-snouted gars [62].

The dentary (De, Figs. 8A, 8D, 9B) occupies the major part of the external surface of the mandible and is ornamented mainly with numerous anteriorly-inclined fine striations and some tubercles in the anterior portion and the ventral edge. The dentary overlaps the angular over a narrow smooth band along the anterior edge of the latter (f.De-Ang, Fig. 8A). And it meets dorsally the surangular (Sang, Figs. 8A, 8D) in a long straight line. Ventrally, the dentaries of both sides suture firmly with each other, forming the anterior part of a fairly long mandibular symphysis (sym, Fig. 9B).

The angular (Ang, Figs. 8A, 8D, 9B) occupies the posteroventral part of the mandible and flares medially to produce a lamina which meets the articular (Art, Fig. 8C) and prearticular (Par, Fig. 8C) in the median side. The ventral division of this lamina extends forward and then meets, and keeps in contact, with its antimere for a relatively long distance (sym, Fig. 9B), thereby lengthening the symphysis which occupies about 45% of the total mandibular length. The involvement of the angular in symphysis is a feature not seen in saurichthyids. The ornament consists mostly of coarse tubercles.

The surangular (Sang, Figs.8A, 8C, 8D, 9B) is so long that its range, clearly defined by the striations on it which are nearly perpendicular to those on the dentary, occupies nearly two thirds of the mandibular length. It carries a row of closely arranged teeth in the oral edge, which extends posteriorly and stops at a rather anterior position, indicating the anterior rim of a long mandibular adductor fossa (add.fs, Figs. 8A, 8C, 9A).

The medial side of the mandible is well exposed in GMPKU-P1359 and P1360 (Figs. 8B, 8C, 9A). The tooth-bearing coronoid (Cor, Figs. 8B, 8C, 9A) is very long. It is highest just in front of the anterior rim of the adductor fossa, and gradually shallows both anteriorly and posteriorly. The coronoids from both sides approach each other anteriorly, and finally meet in the midline and keep in contact for some distance, forming a tooth-bearing mouth floor in this region (Fig. 9A). It is natural to assume that the coronoids of both sides should have formed a depression to receive the downward extrusion of the vomer (Vo, Figs. 3B, 4B, 5A, 7A) to form a pestle-and-mortar structure.

The prearticular (Par, Fig. 8B, 8C, 9A) sutures with the coronoid anterodorsally and the angular posteroventrally. On its surface numerous faint striations radiate from a low ridge parallel to the margin of bone. Near the curve of the ridge, a foramen interrupts the suture between the prearticular and angular (f.VIImd, Fig. 8B, 8C), which is related to the mandibular ramus of the facial nerve.

Remains of the Meckelian bone (Mk, Fig. 8B, 8C) are exposed in the medial side of the mandible in GMPKU-P1359.Their texture gives an impression of fragmentary perichondral linings of that bone.

Although the accurate range of the articular bone (Art, Fig.8A, 8B, 8C) cannot be determined, we can clearly distinguish a transverse glenoid area for receiving the knob of the quadrate (cdy, Fig. 7B_2_). Posteroventral to the condyle is a triangular area, serving for the attachment of the mandibulohyoid ligament (oMHL, Fig. 8D) which is a key structure for lowering the mandibles in the lower actinopterygians [72], [73].

The adductor mandibulae fossa (add.fs, Figs. 8A, 8C, 9A) is extraordinarily long. Its dorsal opening is bounded posteriorly by the articular, medially mostly by the coronoid, laterally by the surangular, and anteriorly by the surangular and coronoid. The anterior margin of the fossa is roughly 60 mm away from the posterior limit of the mandible in GMPKU-P1360. Because the individual size of GMPKU-P1360 is smaller than that of the holotype (P1358), we deduce that the adductor fossa of the holotype must be longer than that in P1360. But on the other side, in the holotype, the distance of the neurocranium is relatively short (only slightly more than 40 mm) between the presumed jaw joint (if at a similar position as in *Saurichthys* [1], i.e., more or less level with the posterior rear of the craniospinal process) and the postorbital wall. Consequently, we assume that in the holotype, when the jaws are in articulation, its long adductor fossa must have extended far forward to the level of the orbitotemporal region and thus reaches or even exceeds the limit of the large fossa (f.am, Figs. 4B, 5A, 5C) in the posterodorsal part of the orbit mentioned above.

#### 2.11 Palatoquadrate

The quadrate part of the palatoquadrate and some remains of relevant dermal bones (the right ectopterygoid) are preserved in GMPKU-P1359.

Both the left and right quadrates (Qu, Figs. 6B, 7A_2_, B) are well preserved with their medial surface exposed. Its dorsal portion is wider and thinner than the ventral. It has a nearly vertical posterior and a concave anterior margin. There is a massive condyle (cdy, Fig. 7B_2_) at the narrow ventral end.

A large part of the concave lateral side of the right ectopterygoid (Ecpt, Fig. 7A_2_) is exposed. Its anterodorsal edge is straight, whereas its hind margin is a rounded notch, which may serve as the anteromedial edge of the adductor foramen of the upper jaw (add.fm, Fig. 7A_2_), comparable to that in saurichthyids [1], [2]. The inner side of ectopterygoid is only partially exposed with a toothed surface.

#### 2.12 Teeth

Teeth are seen at the mouth margins of the jaws, the oral surface of the vomer, the parasphenoid, the coronoid, and the ectopterygoid. Larger teeth are arranged closely in longitudinal rows along the mouth margins of the upper and lower jaws and occasionally along the midline of the vomer (Fig. 10A–E). These teeth are about 0.8 to 1.3 mm wide at the base, and two to three mm high, which are rather small if the large size of the jaws and the skull is taken into consideration. Teeth on the labial side of the mouth margins, the tooth-plate of the parasphenoid, and the median side of the ectopterygoid and coronoid, and most part of the vomer are much smaller.

All teeth are conical with a low enamel cap bearing five to twelve keels radiating from the apex, giving an appearance like the tip of a screwdriver (Fig. 10B, 10D, 10E). This is quite different from the generally sharp teeth with characteristic pointed caps in other saurichthyiforms. Such peculiar structure of the teeth, together with other structural innovations in the feeding mechanism discussed below, points to the likely hard prey-eating habit of the new fish.

#### 2.13 Sensory canals

Passing upward between the paired external nares and then entering the skull roof, the openings of the supraorbital sensory canal (so.c, Figs. 3, 5A) extend posteromedially in the frontal and terminate in the anterolateral margin of the smooth area of the skull roof (sa, Fig. 3A), about at the level of the anterodorsal myodome. Thus, the extension of these openings is more reduced and medially positioned than those in saurichthyids [1], [3], [18]. And these openings are much smaller than those of the sensory canal in the dermopterotic (Dpt, Fig. 3A). The infraorbital sensory canal (io.c, Figs. 3, 5A, 5D, 6A, 7A_2_) in both sides of the snout, after joining the supraorbital canal between the external nares, extends forward along the rostro-premaxilla towards the rostral extremity; however, the anteriormost part has not been clearly seen. There is little information about the suborbital part of the infraorbital sensory canal except a few pores in the nasalo-antorbital and the jugal (Ju, Fig. 6A). More dorsally, the openings of this canal enter the lateral part of the dermosphenotic (Dsph, Figs. 3), and then curve back to the dermopterotic (Dpt, Fig. 3). In the posterior corner of the dermopterotic, there is a tripartite sensory canal which includes the supratemporal commissure (st.c, Fig. 3A) and suggests that the dermopterotic contains a lateral extrascapular component as in saurichthyids [1]. The preopercular and mandibular sensory canals are mentioned above. The mandibular sensory canal is clearly shown in GMPKU-P1360 (mdc, Fig. 9B) running along the lower edge of the mandibles. The preopercular canal can be traced along the vertical ridge (po.c, Fig.7A_2_) in the inner side of the preopercle referred above.

#### 2.14 Dermal pectoral girdle

In the new material, the preserved dermal pectoral girdle includes the paired posttemporals, supracleithra and the right cleithrum, and possibly a clavicle in GMPKU-P1359 (Fig. 6B).

The left posttemporal (Pt, Figs. 6B, 11D) is still attached to the supracleithrum (Scl, Figs. 6B, 11D) without any clear suture between them, whereas the right one is detached from the supracleithrum (Fig. 5B). The posttemporal is roughly triangular with a straight anterior edge originally meeting the dermopterotic, a round and undulating medial, and a sigmoid posterior edge suturing with the supracleithrum. The lateral line is not very clear. Judged from the width of its anterior edge and the width of the skull, the posttemporal may not meet medially with its antimere, which leaves a space for the medial extrascapulars or mid-dorsal scales. The ornament consists of coarse tubercles.

The supracleithrum (Scl, Figs. 6B, 11D) is slightly larger than the posttemporal, roughly trapezoid with the anteroventral margin longest and convex (Fig. 11D). The external surface of this bone is slightly convex with densely arranged fine tubercles and some anastomosing ridges on the middle and posterodorsal portion, and in the latter portion the lateral line is presumably located. On the anteroventral portion, numerous short serrated ridges are seen around the margin with small sawteeth pointing upwards, indicating the area originally overlapped by the opercle when the fish was alive (fa.op, Fig. 11D). Similar ornament is also developed on the opercle-covering area on the cleithrum described below and these characteristic ornamentations are also seen in the comparable region of the dermal pectoral skeleton in saurichthyids [3], [22], [32].

The right cleithrum (Fig. 11A, 11B) is almost completely preserved in GMPKU-P1359 except the dorsal tip of the ascending ramus. It is typically triradiate in shape, similar in structure to that of saurichthyids [1], [2], [4] except the posteroventral process is much longer in proportion. The ventral portion of the cleithrum slightly bends medially. The opercle-covering facet (fa.op, Fig. 11A) is confined within the anterior part of the dorsal ramus and the dorsal portion of the anteroventral process. Similar to the supracleithrum, it is characterized by numerous short serrated ridges with the sawteeth pointing backward and downward, i.e., away from the branchial cavity (Fig. 11A). The remaining area of the cleithrum is decorated with numerous coarse tubercles and fine round pits (Fig. 11A). In the inner side of the bone, three prominent keels emerge from the center of the bone and run in the way shown in Fig. 11B. Interestingly, in the opercle-covering area, at least 13 small pits and two much larger ones left by biting of a predator are discernible (bi.tr, Fig. 11A). The latter are so deep that they have nearly penetrated the bone, causing two swells in the inner side (bi.tr, Fig. 11B) and roughly corresponding to the two distinct holes near the ventral edge of the right opercle (bi.tr, Fig. 11C) in size and position. The biting traces are also seen on the lower jaw (Fig. 8A) of the same specimen. Taken into consideration the preservation of the specimen and no trace of healing for the predating damage, it is likely that the fish was fatally attacked by a huge predator (such as *Birgeria* or large saurichthyid individual).

A triangular plate-like bone is preserved near the left supracleithrum in GMPKU-P1359, probably belonging to the pectoral girdle (Cla?, Figs. 6B, 11E, 11F). The bone has a gently convex, a short concave, and an irregularly undulating borders, for which we labeled ‘a’, ‘b’, and ‘c’ in Fig. 10E, 10F for descriptive reason. One side of the bone (Fig. 11E) is on the whole plane and smooth, with a series of regular frillings in the border ‘a’ and a low ridge along the border ‘c’. On the other side (Fig. 11F), the border ‘a’ is thickened and somewhat sponge-like, whereas the border ‘c’ is slightly undulating with a perpendicular ridge tapering towards the border ‘b’ and extending towards the border ‘a’ as a spongy-like tip. Between this ridge and the thickened part near the border ‘a’ is an obvious channel narrowing towards the angle enclosed by borders ‘a’ and ‘c’, with numerous small foramina in it.

#### 2.15 Other scattered bones

Three scattered smaller bones are preserved in GMPKU-P1359 (Fig. 11G, 11H, 11I). Two of them (Fig. 11H, 11I) are probably lateral line scales. They are triangular and each bears a pore assigned to lateral line canal (f.ll, Fig. 11H, 11I) and some tubercles on the outer surface. The third one (Fig. 11G) is tongue-like with the inner surface exposed, which is much lower along the longitudinal axis than the margins. Its larger end is thickened with some spines on the outer surface, whereas in the other end emerges numerous foramina.

## Discussion

### 1. Phylogenetic analysis of the Saurichthyiformes

Our phylogenetic analysis is aimed mainly at preliminarily evaluating the intrarelationships of the Saurichthyiformes sensu Berg, 1940 [74], and specifically the phylogenetic status of *Yelangichthys*. We are not concerned with the overall lower actinopterygian phylogeny but the Saurichthyiformes' affinity within the Chondrostei sensu Patterson, 1982 [11] and the relationships of the saurichthyiform taxa. Accordingly, our data matrix includes only some relatively well-known saurichthyiforms and other taxa (*Birgeria* (Birgeriiformes sensu Jin, 2001 [75]) and Acipenseriformes) ever considered closely related with the Saurichthyiformes, plus a few taxa as outgroups (*Mimipiscis, Moythomasia*, *Australosomus* and *Amia*).

**Figure 12:**
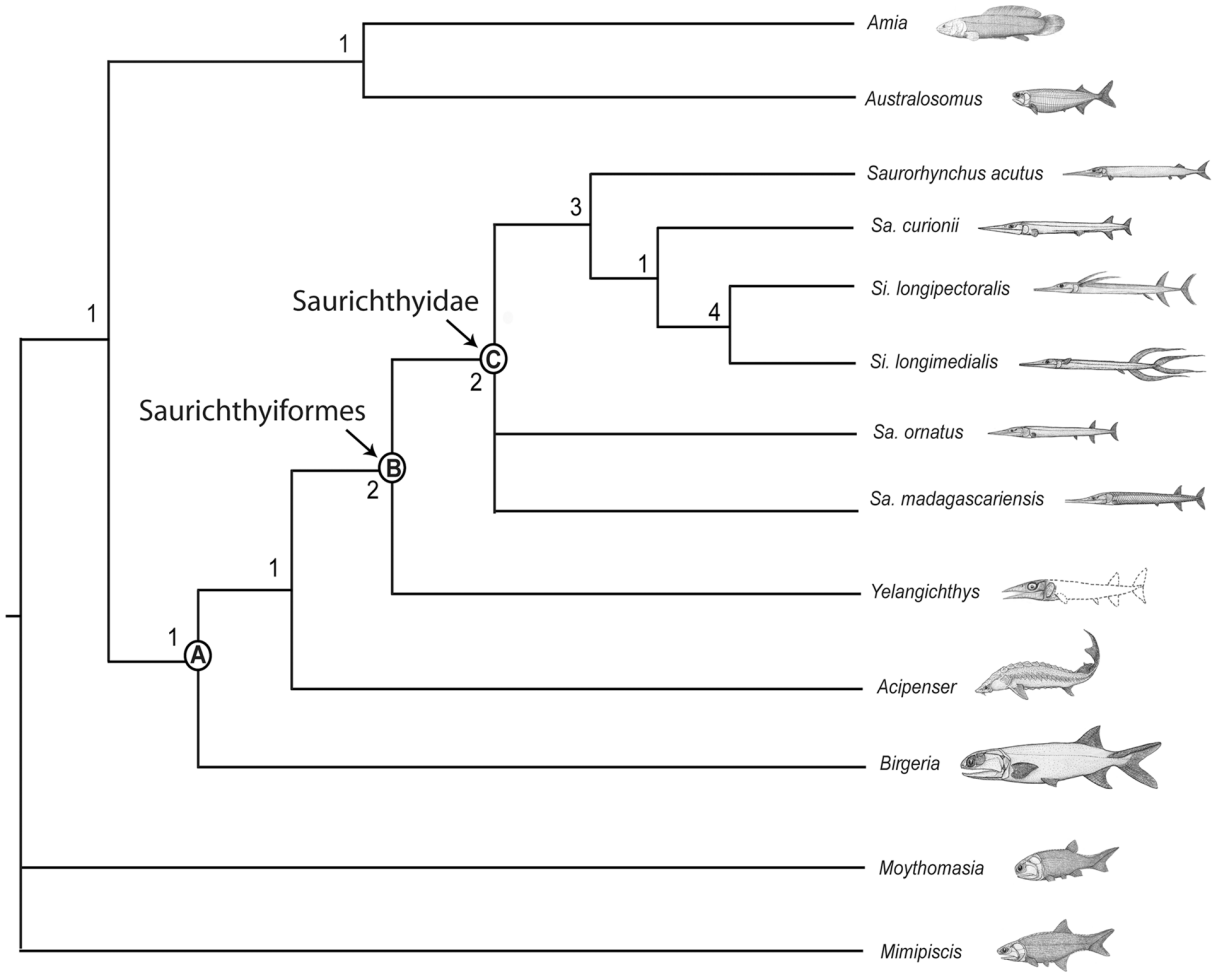
Phylogenetic relationships of *Yelangichthys*. Length  =  122, CI = 0.6885, RI = 0.75. Some nodes indicated by letters. Numerical values near the nodes denote Bremer decay indices. **Abbreviations**: *Sa.* = *Saurichthys*, *Si. = Sinosaurichthys*.

The phylogenetic affinity of Saurichthyiformes (*Saurichthys*) within the lower actinopterygians has been controversial: being either as stem-actinopteran, stem-chondrostean, or even stem-neopterygian [13]–[16]. Its systematic position changes along with the variable definition of the Chondrostei sensu Patterson, 1982. Most recently, Gardiner et al' s [15] and Xu and Gao, 2011's [16] analyses tend to suggest that the Saurichthyiformes is most closely related to the Acipenseriformes within the Chondrostei (*Birgeria* + (Saurichthyiformes + Acipenseriformes)), therefore recognizing a different grouping of the Chondrostei from that in other relevant studies [13], [14]. However, the intrarelationships of Saurichthyiformes have not yet been assessed since Rieppel' s pioneering cladistic work [4] on *Saurichthys* in 1992, which was based on a data matrix of nine species and eight characters. And given the numerous new saurichthyiforms discovered in China [3], especially the new taxon here which displays such complete neurocranial morphology, we here present a phylogenetic analysis to assess the Saurichthyiformes' affinity and to discuss the intrarelationships of saurichthyiforms and the systematic position of the new taxon *Yelangichthys*, based on a dataset composed of 69 characters (including 29 neurocranial ones) coded across four outgroups (Text S1 and Text S2) whose neurocranium has been relatively well investigated (*Mimipiscis* ( = *Mimia* [12], [56]), *Moythomasia*, *Australosomus* and *Amia*) and eight in-group taxa (six well-known saurichthyiforms and two other actinopterygians: *Acipenser* (referring to *Acipenser brevirostris* [76] and *A. ruthenus* [69]) (Acipeneriformes sensu Berg, 1940), *Birgeria* (Birgeriiformes sensu Jin, 2001). The characters were adopted from previous relevant studies (Text S1) and our own observations. Parsimony analysis was conducted using the branch-and-bound algorithm of PAUP v. 4.0b10, with all characters unweighted and unordered. The analysis resulted in two most parsimonious trees of which the strict consensus is shown in Figure 12.

The tree topology agrees with Gardiner et al. [15] and Xu and Gao [16] on the grouping of the Chondrostei (Clade A in Fig. 12), in which the Saurichthyiformes was placed as the sister group of the Acipenseriformes sensu Berg, 1940, in turn these two sister to *Birgeria* (Birgeriiformes sensu Jin, 2001). The synapomorphies supporting the monophyly of the Chondrostei includes: parabasal canal absent; internal carotid artery passing below the parasphenoid; intraosseous dorsal aorta canal absent; well-developed nerve ophthalmicus profundus absent; and supraorbitals present.

Within the Saurichthyiformes (clade B in Fig. 12), *Yelangichthys macrocephalus* (Yelangichthyidae) is resolved as the sister to the Saurichthyidae sensu Stensiö, 1925 [1], and the two constitute the Saurichthyiformes sensu Berg, 1940 (clade B in Fig. 12) based on the following synapomorphies: the orbital artery penetrating parasphenoid behind ascending process; olfactory nerve traversing long distance in orbit; prolonged rostro-premaxilla; discrete nasals, lateral extrascapular bearing tripartite sensory canals and subopercle absent; pronounced posteroventral process of cleithrum present; anteriormost pectoral fin rays unsegmented; caudal fin abbreviated diphycercal; two neural arches of same shape developed in each vertebral segment and the dorsal and ventral roots of the spinal nerve separated by the neural arch. Within this order, *Yelangichthys* (Yelangichthyidae), situated at a basal position, is even more primitive than the Early Triassic saurichthyiforms, i.e., *Saurichthys madagascariensis* and *S. ornatus*, and is excluded from the Saurichthyidae (clade C in Fig. 12) sensu Stensiö, 1925 [1] in having vestigial lateral pillar; olfactory nerve lodged in deeply-cut grove in orbital region; ramus ophthalmicus trigemini and ramus ophthalmicus lateralis separated in orbital region; well-developed nerve ophthalmicus profundus; paired posterior myodomes and distinct pituitary canal. The saurichthyids have a large single posterior myodome for the insertion of the external rectus muscles of both sides and therefore the pituitary canal is obliterated. The following characters also distinguish *Yelangichthys* from saurichthyids: orbitotemporal region distinctly larger than oticooccipital region in proportion; orbital tectum broad, almost reaching lateral margin of dermal skull roof; anterior portion of braincase low, posterior part of vomer bending down abruptly together with deepened braincase; peculiar deep and narrow, transverse fossa in posterodorsal part of orbit; supraorbital(s) absent; dermosphenotic-nasal contact present; denticles delimiting posterior edge of spiracular groove of ascending process of parasphenoid; mandibular adductor foramen in upper jaw and mandibular adductor fossa in lower jaw long, all extending anteriorly to the level of orbit; surangular very long, forming part of tooth-bearing mouth margin in front of adductor fossa; dentary not partaking in adductor fossa; angulars partaking in mandibular symphysis; all teeth caps with screwdriver-like tips.

Among the clade of the Saurichthyidae, all the post-Early Triassic saurichthyid species were grouped together, and separated from the Lower Triassic *Saurichthys madagascariensis* and *S. ornatus*. The post-Early Triassic group herein was supported by the characters as follows: orbital artery penetrating the parasphenoid at the base of ascending process; dermal basipterygoid process developed; efferent pseudobranchial artery penetrating parasphenoid; supraorbital(s) absent; suborbital(s) absent; discrete posttemporal absent; two independent haemal arches in each segment, all bearing haemal spines. As the intermediate taxon between Anisian *Sinosaurichthys* and Lower Jurassic *Saurorhynchus acutus*, *Saurichthys curionii* shares with *Sinosaurichthys* only one synapomorphy: six scale rows. Whereas the grouping of the *Sinosaurichthys* species was better supported: posterior stem of parasphenoid elevated; predorsal mid-dorsal scutes larger than mid-ventral ones; posteroventral process of cleithrum expanded into high plate; pectoral fins inserted dorsally in flank, and elongated; caudal neural spines absent; ossifications of caudal haemal arches in each vertebral unit consisting of one arch bearing haemal spine plus one small intercalary. The relationships of the *Saurichthys madagascariensis* and *S. ornatus* is to be resolved, with one of them being alternatively more basal-ward than the other within the Saurichthyidae (clade C in Fig. 12) in the two most parsimonious trees. Anyway, the phylogenetic distribution of the three *Saurichthys* species (*S. madagascariensis*, *S. ornatus*, and *S. curionii*) selected for current analysis clearly indicates that the type genus of the Saurichthyidae, *Saurichthys* cannot be treated as a monophyletic group (Fig. 12), just as Stensiö predicted that it might be subdivided into several groups [1]. However, the final taxonomic revision is beyond the scope of the current study.

The Neopterygii, diverging from the Chondrostei, shows a set of derived characters: dilatator fossa or depression present; posterior myodome single; pituitary vein canal obliterated by insertion of external rectus muscle; cerebellar corpus undivided; cerebellar arching above fourth ventricle; cerebellar with median anteriorly projecting; epibranchial I and II with strongly forked ends; caudal fin hemi-heterocercal with elongated upper rays.

### 2. Endoskeletal variations of the neurocranium

Prior to the discovery of the *Yelangichthys*, the saurichthyiform neurocranium is rather consistent morphologically, showing little differences during their history from the Early Triassic to Early Jurassic [1], [17]–[19], [38]. However, the addition of *Yelangichthys* increases many endocranial variations which concerns the breadth of the orbital tectum (along with the width of the skull roof table), the peripheral arrangement of some nerves, the structure of the posterior myodome, and the presence of a unique deep fossa in the posterodorsal part of the orbit.

The orbital tectum of the saurichthyids is, as usually the case in most known bony fishes [1], [12], [17], [36], [37], [46], [47], [53]–[55], relatively narrow and always constricts medially at the level of the center of the orbit. Consequently, the interorbital part of the tectum is much narrower than the dermal skull roof. But in sharp contrast, it is so broad in *Yelangichthys* that it extends laterally nearly as much as the dermal bones do, thereby forming a large endoskeletal roof for the orbit, and thus makes it possible to trace the courses of some vessels and nerves in this region, which were lodged or contained in the grooves or canals in the underside of the tectum. For example, the ramus ophthalmicus lateralis innervating the supraorbital canal and ramus ophthalmicus superfacialis trigemini for other sensory organs in the skin were lodged in the same canal and groove, i.e., running along together with each other, a similar state shared by most other bony fishes [1], [12], [21], [37], [53], [64] and thus being plesiomorphic. Instead, these two rami were distinctly separated along their courses in saurichthyids [1] and extant acipenseriforms, e.g., *Acipenser* and *Polyodon* [1].

As pointed out by Schaeffer and Dalquest [57], there is a transformation series of the posterior myodome from being absent, via paired, to eventually single in the evolutionary history of the actinopterygians. The posterior myodome was not developed in most basal actinopterygians, such as the *Mimipiscis* ( = *Mimia*) [12], [56] and *Polypterus* [54]; and later, along with the increasing length of the external recti muscles, a pair of myodomes appeared in some more derived forms, e.g., *Myothomasia* [12] and *Kentuckia* [77], and eventually, a single myodome emerged in the more advanced taxa. Interestingly, with the discovery of *Yelangichthys*, such a transformation partially recurs within saurichthyiforms from the paired myodomes to a single one in saurichthyids.

Different from in other lower actinopterygians in which the course of the olfactory nerve is usually continuous as a bone-enclosed canal between the nasal and the cranial cavities [12], [36], [37], [43], [48], [50]–[55], [63]–[69], the olfactory canal was interrupted in the orbital region to a relatively great extent in saurichthyiforms. However, there are variations in the specific arrangement across members. In saurichthyids, the olfactory nerve, before entering the postnasal wall via the ethmoid olfactory canal, extended in the orbit leaving no traces on the interorbital wall [1]. But this nerve in *Yelangichthys* was lodged in a deep groove in the interorbital wall, and the opening of the ethmoid olfactory canal is more posteriorly located than that in saurichthyids, i.e., posterior, instead of anterior, to the anterodorsal myodome.

Most striking structure of the neurocranium is the transverse deep fossa in the posterodorsal part of the orbit, which opens ventrally and is so deep that it has nearly penetrated the endocranium. No comparable fossa has ever been documented before either in other saurichthyiforms or other known fossil bony fishes [1], [12], [14], [17]–[19], [21], [33], [36]–[38], [40], [43], [46]–[53], [55], [57], [58], [60], [62], [65]–[67], [69], [77], although it is reminiscent of the supraorbital fontanelle housing some adductor muscle in *Polypterus* [54] and the arrangement that some adductor muscle originates upon the neurocranium in the orbit in *Latimeria* [78], [79]. We assume this unique fossa in *Yelangichthys* should be related with the peculiar arrangement of the mandibular adductor musculature, if its feeding mechanism as a whole is taken into consideration (see discussion below).

### 3. Functional innovations in the feeding mechanism and a new dietary preference

Several aspects of the feeding mechanism of *Yelangichthys* point to durophagy, a new feeding adaptation among saurichthyiforms: 1) small and crushing-type teeth; 2) hypertrophied adductor muscle arranged in high mouth-closing mechanical advantage; 3) relatively large suspensorium angle and large volume of mouth cavity; 4) long mandibular symphysis.

Saurichthyiforms are usually considered as fierce fish-eaters evidenced by stomach contents of fish skeletal relics, hunting like the extant gars or pikes [2], [4], [19], [29]. Recently, another lifestyle has been assumed for *Siniosaurichthys*, a likely surface cruiser leading a life similar to that of living needlefishes [3]. However, the dietary preference of all of these forms is typically piscivorous, indicated by their sharp marginal teeth. Conversely, the teeth of *Yelangichthys* are of crushing type rather than piercing one in saurichthyids (Fig. 10F). Additionally, the teeth are too small to grasp. These dental features suggest that *Yelangichthys* is likely best used to durophagy, consuming some shelly animals, such as thin-shelled or tiny bivalves discovered from the same fauna [27].

**Figure 13:**
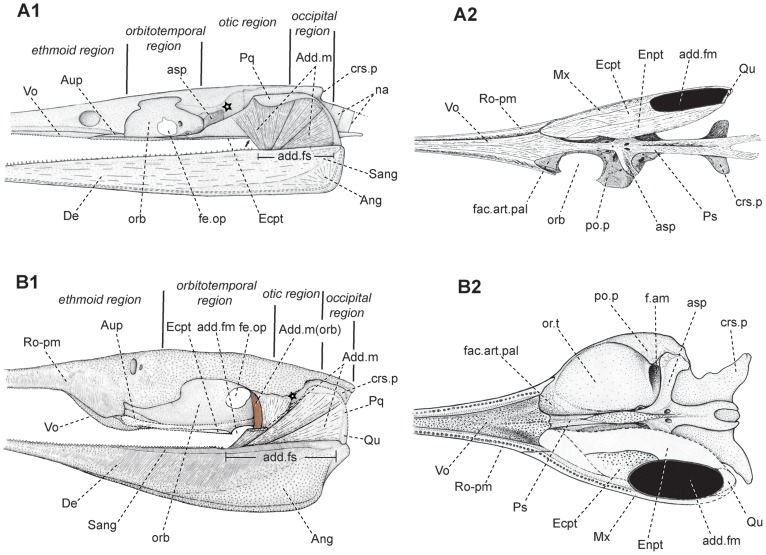
Restoration of jaw mechanism of *Saurichthys* and *Yelangichthys*, based on [1], [37], [54], [72], [73], [79], [83]. **A1** (*Saurichthys*), **B1** (*Yelangichthys*), in lateral view with check bones removed; **A2** (*Saurichthys*), **B2** (*Yelangichthys*), in palatal view; Anterior facing left. Arrow in **A1** pointing the anterior rim of adductor foramen in upper jaw, and asterisks indicating the center of hyomandibular facet. **Abbreviations**: **Add.m (orb)**, adductor mandibulae muscle originating in orbit; **Add.m**, adductor mandibulae muscles; **Aup**, autopalatine; **orb**, orbit. See Figs.3–5, 7–9 for other abbreviations.

Such an assumption is also supported by the arrangement of the mandibular adductor muscle. To show this, we model the lower jaw as a lever, in which the jaw joint is the fulcrum, and the adductor muscle supplies the input and the mouth-closing force transferred to the teeth to produce bite force. For durophagy, high mouth-closing advantage is necessary [80], which is determined by the force of the adductor muscle and the input arm. Generally speaking, the force of a muscle is directly proportional to its cross sectional area [80]. Specifically, for the adductor muscle, the length of its insertion area (adductor fossa) is a proxy for the cross sectional area, and thus its size and strength. The adductor fossa in *Yelangichthys* is so long that it occupies nearly one third of the overall mandibular length and is proportionally much longer than that in saurichthyids (Fig. 13A_1_, A_2_). This indicates a hypertrophied adductor muscle (Fig. 13B_1_), which has the potential to generate greater mouth-closing power in *Yelangichthys* than in saurichthyids, such as *Saurichthys* (Fig. 13A_1_) and *Sinosaurichthys* [3]. At the same time, due to the prolonged adductor fossa (insertion area), the input arm (measured from the jaw joint to the center of the adductor fossa according to Anderson et al. [80]) is consequently increased. On the other hand, judged from the structure of the maxilla and palatoquadrate (ectopterygoid) which involve in the construction of the adductor foramen in the upper jaw, this foramen must be also very long (add.fm, Fig. 13B_2_), corresponding to the elongate adductor fossa in the lower jaw. Therefore we assume that *Yelangichthys* has a considerable functional advantage to close the mouth via a relatively great input moment (i.e., input force by input arm).

Another relevant point concerns the increase of the mandibular symphysis (sym, Fig. 9B_2_). As is known, the relative size of the mandibular symphysis will affect how well it can withstand certain shear stresses and torsion during chewing [80], [81], so the increased symphysis in *Yelangichthys* means better ability to take on such a function. On the other hand, this innovation is necessary, if the structure of the mouth is taken into consideration. In the mouth roof there is a conspicuous bulge in the posterior ethmoid region (as the arrows point in Figs. 3B, 5A, 7A_2_), and it is not difficult to assume the existence of a corresponding depressed part in the mouth floor to form a pestle-and-mortar like structure to crush or crack preys. To meet the functional demand, it is optimized for the mandibular symphysis to extend to this level.

Additionally, the relatively large suspensorium angle of *Yelangichthys* is also a significant feature, because it is related to the manner of the jaws suspension and the volume of the mouth cavity [72], [73], [82], [83]. According to Gardiner et al. [15] this angle can be estimated largely by the position of the jaw joint in relation to the hyomandibular facet. Here in *Yelangichthys*, the reduced length of the postorbital part of the neurocranium and the cheek bones, and the nearly vertical posterior border of the preopercle make the jaw joint more anteriorly situated in relation to the hyomandibular facet and thus form a larger suspensorium angle. As estimated in Gardiner et al.' s method, this angle of *Yelangichthys* has more or less doubled that gauged in *Saurichthys ornatus* (ca. 40°*vs* 20°). This means that the suspensorium of *Yelangichthys* is more vertically oriented than in ‘general’ saurichthyids which possess a proportionally longer cheek region resulting in a more obliquely arranged suspensorium, such as the Early Triassic species of *Saurichthys* [1]. This morphological change, together with the hypertrophied adductor muscle forming the lateral walls of the mouth cavity, substantially increases the volume of the mouth cavity which involves generating minus pressure to suck in preys [72], [73]. Since such an increase is at the expense of the mouth gape, it is good for the durophagous animal like *Yelangichthys* to transport the cracked prey items into the mouth cavity and reduce the odd of losing food items through the gape.

All of the innovations summarized above of the feeding mechanism of *Yelangichthys* point to a durophagous feeding strategy for saurichthyiforms. *Yelangichthys* would not have been an efficient fish-eater and instead more likely was a durophagous fish consuming shelled animals.

It is also interesting that such a functional and trophic variation appeared during the Anisian in the east Tethys, when and where saurichthyiforms were taxonomically so highly diversified [3], [22]. A possible interpretation for this coincidence is that this is a special stage of adaptive radiation after the fatal end-Permian extinction [23], [24], [84]. Saurichthyiforms had exploited different food resources, which resulted in not only the sheer survival but also a marked taxonomical diversity and ecological differentiation of the group.

## Supporting Information

Text S1 **Character list.**
(DOCX)

Text S2 **Data matrix for phylogenetic analysis (Abbreviations** ***Sr*** **.:** ***Saurorhynchus*** **;** ***Sa*** **.:** ***Saurichthys*** **;** ***Si*** **.:** ***Sinosaurichthys*** **).**
(DOCX)
