# Supplementary material for: A New Saurichthyiform (Actinopterygii) with a Crushing Feeding Mechanism from the Middle Triassic of Guizhou (China)
Source: PLoS One. 2013 Dec 4;8(12):e81010. doi: 10.1371/journal.pone.0081010 (PMC3852010; doi:10.1371/journal.pone.0081010)
Supplement: Text S1 — Character list. (DOCX) [file pone.0081010.s001.docx]

**Text S1. Character list.**

The characters are partially adopted from previous publications on the phylogenetic relationships of lower actinopterygians (GS, Gardiner and Schaeffer, 1989 [1]; R, Rieppel, 1992 [2]; C, Coates, 1999 [3]; CA, Cloutier and Arratia, 2004 [4]; GSM, Gardiner et al., 2005 [5]; G, Grande, 2010 [6]; XG, Xu and Gao, 2011[7]).

1. Posttemporal fossa:(0) absent; (1) present. (modified from GS28; C33; GSM1; XG1; G32)

2. Fossa Bridgei: (0) absent; (1) present. (XG5; modified from C31)

3. Dilatator fossa or depression: (0) absent; (1) present. (GS31; C34; GSM2; XG5)

4. Lateral pillar or its vestige: (0) present; (1) absent.

According to Jarvik [8], the lateral pillar in *Amia* is developed in early embryonic stage and represents the visceral suprapharyngomandibular component of the mandibular arch, which partakes in the neurocranium as a strut separating the trigeminofacialis chamber and the posterior myodome and dividing some relevant nerves and vessel into several groups [8,9]. The comparable structure of this pillar or its vestige is present in a great variety of actinopterygian neurocrania, which is termed as alisphenoid (pterosphenoid) pedicle in diverse taxa ranging from the Devonian to present, such as *Moythomasia* [10], *Kansasiella* [8,11], *Birgeria* [12], *Sinamia* [13] and some other more derived neopterygians [14], but it is not developed in *Polypterus*, *Acipenser* and *Lepisosteus* [8,9], nor in *Pteronisculus* ([15], contra [16]) or saurichthyids [17,18,19]. Although the homology of the lateral pillar in actinopterygians with the postorbital pillar (also termed as lateral pillar in [20]) in some primitive osteichthyan and sarcopterygian (e.g. *Guiyu* [21], *Psarolepis* [20,22], *Archoania* [23] and *Styloichthys* [24]), and the postocular lamina in the arthrodirian ‘placoderm’ [25,26]) is uncertain yet, based on their similar topological relations to the adjacent structures, such as the jugular vein, basipterygoid process, posterior myodome (where present) and trigeminofacialis chamber or the space housing trigeminal and preotic lateral ganglia, it appears to be more parsimonious to consider those structures homologous, if the developmental framework of Stensiö and Jarvik on the early vertebrate skull is accepted. Additional support for this argument may come from the neurocrania of a galeaspid *Shuyu* [27,28], where the dorsal most portion of the mandibular arch straddles the jugular vein and is situated between the postventral myodome (myodome for VI-innervated eye muscle, i.e., posterior myodome of osteichthyans) and trigeminal recess, again a similar configuration as in *Amia* [8]. Therefore, judged from comparisons above, the presence of this pillar or its vestige is a primitive feature for actinopterygians, just as Patterson stated [14].

5. Parabasal canal: (0) present; (1) absent. (XG10)

Parabasal canal is the tube formed by the dermal parasphenoid ventrally and the floor of the neurocranium dorsally, and the tube lodges the palatine nerve along with the internal carotid artery and its anterior branch, the palatine artery [29]. This canal was first referred to by Allis [30] in *Amia* and first termed as the parabasal canal by Gaupp [31]. *Australosomus* does not possess such a canal according to Nielsen [32], contra being present in Xu and Gao' s dataset [7].

6. Internal carotid artery extending below parasphenoid: (0) absent; (1) present. (modified from GSM11)

In the vast majority of actinopterygians, before entering the basicranium, the internal carotid artery always extends either through the interspace between the cranial base and the parasphenoid corpus, or through the bone-enclosed canal in this region, i.e., above the parasphenoid [8,10,11,14,17,33] except in some fossil taxa: *Birgeria* [34] and *Saurichthys* [17] and *Yelangichthys* herein, and also the extant acipenseriforms *Acipenser* [17,35] and *Polyodon* [17,36], where this artery extends laterally or even ventrally to the parasphenoid, a situation probably related in part to the absence of the circulus cephalicus [37].

7. Penetration of orbital artery in parasphenoid: (0) absent; (1) behind ascending process of parasphenoid; (2) at the base of ascending process of parasphenoid; (3) anterior to ascending process of parasphenoid.

8. Dermal basipterygoid process: (0) absent; (1) present. (C37; modified from GSM12; coding 1 for *Sauroryhnchus* based on FX Wu pers. observ. of BMNH P.36221 and P.36226)

9. Well-developed craniospinal processes supporting the dermal skull roof: (0) absent; (1) present. (modified from GSM7 and XG13)

10. Confluence of notochord canal with ventral otic fissure (regression of notochord, see discussion in Hamel and Poplin, 2008 [33]): (0) absent; (1) present.

11. Bifurcation of dorsal aorta in: (0) otic region; (1) occipital region or more posterior position.

The dorsal aorta bifurcates into paired lateral dorsal aortae in the occipital region or even more posteriorly in primitive sarcopterygians *Griphognathus* [38], *Eusthenopteron* [8], *Youngolepis* [39], and also in the extant coelacanth *Latimeria* [40]. This is also the case for the stem-group gnathostomes antiarch [41] and arthrodiran [25] ‘placoderms’. And the developmental process of *Amia* also displays an anterior disposition of this bifurcation point [8]. Therefore, the posteriorly bifurcating of the dorsal aorta is plesiomorphic for actinopterygians whereas the more anterior bifurcation pattern in some basal actinopterygians, such as *Mimipiscis* and *Moythomasia* [10], should be considered as a derived specialization [42] rather than a primitive feature as Coates claimed [15]. Also, the evolution of the epibranchial arterial system and the variation of the bifurcation point of dorsal aorta in actinopterygians seem to be unnecessarily linked with the length of the bone-enclosed dorsal aorta canal and that of the posterior part of the parasphenoid [42].

12. Intraosseous dorsal aorta canal: (0) present; (1) absent. (modified from C30)

13. Arrangement of the olfactory nerve in orbital region: (0) enclosed in osseous olfactory canal; (1) traversing

in orbit without any traces on the interorbital wall; (2) lodged in short but deep groove.

In most osteichthyans, including the basal taxa *Ligulalepis* [43], *Psarolepis* [20,22], and the primitive sarcopterygians, such as *Diplocercides* [8,44,45], *Styloichthys* [20,24], *Youngolepis* [39], *Griphognathus* [38-91] and *Eusthenopteron* [8-35] and most known actinopterygian taxa, the olfactory nerve always extends in the bone-enclosed canal within the interorbital wall [8,10,11,14,16,32,33] except in saurichthyids [17,46,18] and *Yelangichthys* here. For saurichthyids, this nerve passes through in the orbit for a considerable distance, leaving no traces on the interorbital wall [17], whereas in *Yelangichthys* it is lodged in a deep and relatively short groove in the interorbital wall.

14. R. (= ramus) opth. (= ophthalmicus) trigemini and r. ophth. lateralis: (0) convergent; (1)

divergent.

These two rami are usually closely associated in most fishes [17], both in fossil and living forms [8, 10,11,16,33], but they extend forward far apart in *Saurichthys* [17], *Acipenser* [17,35] and *Polyodon* [17]; however, they are closely arranged along their courses in *Yelangichthys* here.

15. Well-developed nerve ophthalmicus profundus: (0) present; (1) absent.

The nerve ophthalmicus profundus is always well developed and terminates in the nasal capsule in most fishes, but it is difficult to ascertain the existence of this nerve in *Acipenser* and *Polyodon* [17] or so less developed that it probably did not extend as forward as the ethmoidal region in *Saurichthys* [17]. In contrast to the absence of the canal for this nerve in some *Saurichthys* species [17], in *Yelangichthys* the opening of the profundus canal is seen in its usual position somewhat medial to the lateral pillar [8].

16. Penetration of efferent pseudobranchial artery in parasphenoid: (0) absent; (1) present.

17. Elevation of posterior stem of parapshenoid: (0) absent; (1) present.

18. Downward bulging vomer and ethmoidal region of neurocranium: (0) absent; (1) present.

19. Denticles delimiting posterior edge of spiracular groove: (0) absent; (1) present.

20. Posterior myodome: (0) absent; (1) paired; (2) median. (modified from GSM6, XG7)

21. Pituitary vein canal: (0) present; (1) absent. (modified from C38)

22. Pituitary canal obliterated by insertion of external rectus muscle: (0) absent; (1) present. (modified from C38)

23. Roof of posterior myodome perforated by r. VIIpal. : (0) absent; (1) present.(modified

from C39)

24. Shape of orbital tectum at interorbital level: (0) concave medially; (1) convex laterally.

25. Ratio of orbitotemporal to otico-occipital regions of braincase: (0) smaller or nearly equal in length; (1) distinctly larger and longer.

26. Large fossa in posterodorsal corner of orbit: (0) absent; (1) present.

27.Cerebellar corpus: (0) divided bilaterally; (1) undivided. (C50; XG70)

28.Cerebellar and fourth ventricle: (0) cerebellar corpus entering in fourth ventricle; (1) the former arching above the latter. (C51; XG71)

29. Cerebellar with median anteriorly projecting: (0) absent; (1) present. (C52; XG72)

30. State of squamation: (0) complete squamation; (1) six scale rows; (2) four scale rows; (3) trunk naked but with caudal scales; (4) trunk with scale rows and caudal scales. (modified from GSM35)

31. Size relation of predorsal scutes to mid-ventrals: (0) similar in size; (1) distinctly larger.

A feature of *Sinosaurichthys* [47] which indicates the special body profile: epaxial portion broader than the hypaxial and is associated with their dorsally-inserted pectoral fins.

32. Posterior external narial involvement of anterior orbital border: (0) present; (1) absent.

33. Discrete nasal bones: (0) present; (1) absent. (CA43; XG23)

34. Supraorbital (s): (0) absent; (1) present. (C7, GSM22, XG26, R3)

35. Dermosphenotic-nasal contact (dermosphenotic spanning the dorsal rim of orbit): (0) present; (1) absent. (C6,

XG18)

36. Discrete lateral extrascapular: (0) present; (1) absent.

The supratemporal commissure traverses the extrascapular series with the trifurcate point of the sensory canals in the discrete lateral extrascapular in most occasions but not in saurichthyiforms where the lateral extrascapulars is fused with the dermopterotic, making the joint of the commissure with the lateral line within a compounded ‘dermopterotic’ [17].

37. Discrete intertemporal: (0) present; (1) absent. (modified from GSM17, C4, XG17, R2)

38. Median meeting of dermopterotics: (0) absent; (1) present. (modified from GSM17)

39. Suborbital(s): (0) absent; (1) present (C8, modified from GSM23).

40. Subopercle: (0) smaller than opercle; (1) as the main gill cover without opercle; (2) absent.

41. Fewer than 10 branchiostegal rays: (0) absent; (1) present. (modified from C12)

42. Fringing fulcra: (0) present; (1) absent. (XG50, modified from GSM34)

43. Termination of supraorbital canal in: (0) parietal; (1) frontal; (2) dermopterotic.

44. Prolonged rostro-premaxilla into acute snout: (0) absent; (1) present.

45. Arrangement of labial teeth: (0) large teeth intervened with numerous smaller ones; (1) teeth arranged closely without intervening smaller ones.

46. Shape of enamel cap of teeth: (0) sharp, conical; (1) blunt, like screw-driver tip.

47. Anterior extension of adductor foramen of upper jaw to level of orbit: (0) absent; (1) present.

48. Anterior extension of mandibular adductor fossa to level of orbit: (0) absent; (1) present.

49. Size of surangular: (0) short; (1) long.

50. Partaking of dentary in adductor fossa: (0) present; (1) absent.

51. Partaking of coronoid in adductor fossa: (0) absent; (1) present.

52. Angular partaking in mandibular symphysis: (0) absent; (1) present.

53. Medial contact of prearticular with angular: (0) absent; (1) present.

54.Maxilla free from preopercle: (0) absent; (1) present. (C15; XG31)

55. Epibranchial I and II with strongly forked ends (uncinate process): (0) absent; (1) present. (C25; XG42)

56. Discrete posttemporal: (0) present; (1) absent.

57. Posteroventral process of cleithrum: (0) absent; (1) low and short; (2) low and long; (3) expanded into high plate.

58. Insertion of pectoral fin in flank: (0) ventral; (1) dorsal.

59. Unsegmented anteriormost pectoral fin rays: (0) absent; (1) present.

60. Elongation of fins: (0) no fins elongated; (1) pectoral fins elongated; (2) median fins elongated.

61. Caudal fin: (0) heterocercal without elongated upper rays; (1) abbreviated diphycercal; (2) hemi-heterocercal with elongated upper rays. (modified from C57, XG63)

62. Number of fin rays equals to supporting radials: (0) absent; (1) present. (C55)

63. Caudal neural spines: (0) present; (1) absent.

64. Median caudal neural spines: (0) absent; (1) present. (C56 and GSM33; coding 1 for *Birgeria* based on pers. observ. of FX Wu on IVPP P12569 (*Birgeria liui*) [48]) and BMNH P19286, 26447, 26448)

65. Shape of neural spine: (0) spine-like; (1) plate-like.

66. Separation of dorsal and ventral roots of spinal nerve in same segment by neural arch: (0) absent; (1) present.

Generally in fishes, the ventral motor and dorsal sensory roots of the spinal nerve within the same segment exit from the spinal cord through the interspace between the neighboring neural arches [8,49,50], or between the neural arch and the successive small intercalary element (‘interdorsals’ in some previous literatures, the old terminology according to Gadow´s arcualia theory, which has been questioned by [51,52]), or even separated by the intercalary elements [8,12,35,49], with the exception of saurichthyids in which each segment has two neural arches of similar structure, with one of them separating the ventral and dorsal roots of one spinal nerve just as Stensiö proposed [17]. This is probably a design functionally significant for supporting and stiffening such an elongated body of this group (more detailed discussion in our forthcoming paper),

The following Character 67, 68 are also directly associated with the specialization of saurichthyids referred above.

67. Two neural arches of same shape in one vertebral unit: (0) absent; (1) present.

68. Ossifications of caudal haemal arches in one vertebral unit: (0) one arch bearing haemal spine plus one small intercalary; (1) only one arch bearing spine; (2) one large haemal element without spine, corresponding to two neural arches; (3) two arches, all bearing spines.

69. Shape of haemal spine: (0) spine-like; (1) plate-like.

**References**

1. Gardiner BG, Schaeffer B (1989) Interrelationships of lower actinopterygian fishes. Zool J Linn Soc 97: 135–187.

2. Rieppel O (1992) A new species of the genus *Saurichthys* (Pisces: Actinopterygii) from the Middle Triassic of Monte San Giorgio (Switzerland), with comments on the phylogenetic interrelationships of the genus. Palaeontographica (A) 221: 63–94.

3. Coates MI (1999) Endocranial preservation of a Carboniferous actinopterygian from Lancashire, UK, and the interrelationships of primitive actinopterygians. Philos Trans R Soc Lond B 354: 433–462.

4. Cloutier R, Arratia G (2004) Early diversification of actinopterygians. In: Arratia G, Wilson MVH, R. Cloutier R, editors. Recent Advances in the Origin and Early Radiation of Vertebrates. München: Verlag Dr. Friedrich Pfeil. pp. 217–270.

5. Gardiner BG, Schaeffer B, Masserie AJ (2005) A review of the lower actinopterygian phylogeny. Zool J Linn Soc 144: 511–525.

6. Grande L (2010) An empirical synthetic pattern study of gars (Lepisosteiformes) and closely related species,

based mostly on skeletal anatomy: The resurrection of holostei. American Society of Ichthyologists and

Herpetologists Special Publication, Copeia 10: 1–871.

7. Xu GH, Gao KQ (2011) A new scanilepiform from the Lower Triassic of northern Gansu Province, China, and phylogenetic relationships of non-teleostean Actinopterygii. Zool J Linn Soc 161: 595–612.

8. Jarvik E (1980) Basic Structure and Evolution of Vertebrates. Vol. I, II. London: Academic Press. 575 p.

9. De Beer GR (1937) The development of the vertebrate skull. Oxford: Clarendon Press. 552 p.

10. Gardiner BG (1984) The relationships of the palaeoniscid fishes, a review based on new specimens of *Mimia* and *Moythomasia* from the Upper Devonian of Western Australia. Bull Br Mus Nat Hist (Geol) 37: 173–428.

11. Poplin C (1974) Étude de quelques paléoniscidés pennsylvaniens du Kansas. Cahiers de Paléontologie. Paris: C.N.R.S. 151 p.

12. Stensiö EA (1921) Triassic Fishes from Spitzbergen, Part I. Vienna: Adolf Holzhausen. 307 p.

13. Stensiö EA (1935) *Sinamia zdanskyi*, a New Amiid from the Lower Cretaceous of Shantung, China. Palaeont Sin C3: 1-48.

14. Patterson C (1975) The braincase of pholidophorid and leptolepid fishes, with a review of the actinopterygian braincase. Philos Trans R Soc Lond B 269: 275–579.

15. Coates MI (1998) Actinopterygians from the Namurian of Bearsden, Scotland, with comments on the early evolution of actinopterygian neurocrania. Zool J Linn Soc 122: 27-59.

16. Nielsen E (1942) Studies on Triassic fishes from East Greenland. I. *Glaucolepis* and *Boreosomus*. Meddr Grønland 138: 1-403.

17. Stensiö EA (1925) Triassic Fishes from Spitzbergen, Part II. Kgl sv VetAkad Handl (ser. 3) 2: 1–261.

18. Gardiner BG (1960) A review of certain actinopterygian and coelacanth fishes, chiefly from the Lower Lias. Bull Br Mus Nat Hist (Geol) 4: 239–384.

19. Thies D (1985) Funde von *Acidorhynchus brevirostris* (Woodward 1895) aus dem Posidonienschiefer (Unter−Toarcium) NW−Deutschlands. Palaeontographica (A) 187: 183–203.

20. Yu XB (1990) Cladistic analysis of sarcopterygian relationships with a description of three new genera of Porolepiformes from the Lower Devonian of E. Yunnan, China. PhD Dissertation, Yale University, New Haven. 317 p.

21. Qiao T, Zhu M (2010) Cranial morphology of the Silurian sarcopterygian *Guiyu oneiros* (Gnathostomata: Osteichthyes). Sci China Earth Sci 53: 1836-1848.

22. Yu XB (1998) A new porolepiform-like fish, *Psarolepis romeri*, gen. et sp. nov. (Sarcopterygii, Osteichthyes) from the Lower Devonian of Yunnan, China. J Vert Paleont 18: 261-274.

23. Zhu M, Yu XB, Ahlberg PE (2001) A primitive sarcopterygian fish with an eyestalk. Nature 410: 81-84.

24. Zhu M, Yu XB (2002) A primitive fish close to the common ancestor of tetrapods and lungfish. Nature 418: 767-770.

25. Stensiö EA (1963) Anatomical studies on the arthrodiran head. 1. Preface, geological and geographical distribution, the organization of the arthrodires, the anatomy of the head in the Dolichothoraci, Coccostemorphi and Pachyostemorphi. Taxonomic appendix. Kgl sv VetAkad Handl 9 (2): 1-419.

26. Piveteau J (1969) Traité de Paléontologie. Tome 4, Vol. 3. Paris: Masson. 790 p.

27. Gai ZK, Donoghue P CJ, Zhu M, Janvier P, Stampanoni M (2011) Fossil jawless fish from China foreshadows early jawed vertebrate anatomy. Nature 476: 324-327.

28. Gai ZK (2012) The cranial anatomy of Galeaspida (Agnatha) and the origin of jawed vertebrates. PhD Dissertation, University of Bristol. 453 p.

29. De Beer GR (1926) Studies on the vertebrate head. II. The orbito-temporal region of the skull. Q J Microsc Sci 70: 263-370.

30. Allis EP (1897) The cranial muscles and cranial and first spinal nerves in *Amia calva*. J Morph 12: 487-808.

31. Gaupp E (1900) Das Chondrocranium von Lacerta agilis, AnatHefte 15: 435-594.

32. Nielsen E (1949) Studies on Triassic fishes from East Greenland II. *Australosomus* and *Birgeria*. Meddr Grønland 146: 1-309.

33. Hamel M-H, Poplin C (2008) The braincase anatomy of *Lawrenciella schaefferi,* actinopterygian from the Upper Carboniferous of Kansas (USA). J Vert Paleont 28: 989–1006.

34. Schwarz W (1970) Die Triasfauna der Tessiner Kalkalpen. XX. *Birgeria stensiöi* Aldinger. Schweiz Paläont Abh 89: 1-93.

35. Marinelli W, Strenger A (1973) Vergleichende Anatomie und Morphologie der Wirbeltiere. IV. Lieferung. Wien: F. Deuticke. pp. 309-460.

36. Danforth CH (1912) The heart and arteries of *Polyodon*. J Morph 23: 409-454.

37. Gardiner BG (1973) Interrelationships of teleostomes. In: Greenwood PH, Miles RS, Patterson C, editors. Interrelationships of fishes. London: Academic Press. pp. 105-135.

38. Miles RS (1977) Dipnoan (lungfish) skulls and the relationships of the group: a study based on new species from the Devonian of Australia. Zool J Linn Soc 61: 1-328.

39. Chang MM (1982) The braincase of *Youngolepis*, a Lower Devonian crossopterygian from Yunnan, south-western China. PhD Dissertation, University of Stockholm. 113 p.

40. Millot J, Anthony J, Robinneau D (1978) Anatomie de *Latimeria chalumnae*. Tome III. Appareil digestif, appareil respiratoire, appareil urogénital, glandes endocrines, appareil circulatoire, teguments, ecailles, conclusions generals. Paris: C.N.R.S. 198 p.

41. Young GC (1980) A new Early Devonian placoderm from New South Wales, Australia, with a discussion of placoderm phylogeny. Palaeontographica (A) 167: 10-76.

42. Poplin C (1975) Remarques sur le système artériel épibranchial chez les actinoptérygiens primitifs fossiles. Colloques Internationaux. Paris: C.N.R.S. 218: 265-271.

43. Basden AM, Young GC (2001) A primitive actinopterygian neurocranium from the Early Devonian of southeastern Australia. J Vert Paleont 21: 754-766.

44. Stensiö EA (1937) On the Devonian coelacanthids of Germany with special reference to the dermal skeleton. Kgl sv VetAkad Handl, Ser. 3, 16(4): 1-56.

45. Forey PL (1998) History of the coelacanth fishes. London: Chapman and Hall. 419 p.

46. Beltan L (1968) La faune ichthyologique de l’Eotrias du N.W. de Madagascar le neurocrâne. Paris: C.N.R.S. 135 p.

47. Wu FX, Sun YL, Xu GH, Hao WC, Jiang DY, et al. (2011) New saurichthyid actinopterygian fishes from the Anisian (Middle Triassic) of southwestern China. Acta Palaeontol Pol 56: 581-614.

48. Jin F (2001) Notes on the discovery of *Birgeria* in China. Vert PalAsiat 39: 168-176.

49. Goodrich ES (1930) Studies on the structure and development of vertebrates. London: Macmillan. 837 p.

50. Arratia G, Schultze HP, Casciotta J (2001) Vertebral column and associated elements in Dipnoans and comparison with other fishes: development and homology. J Morph 250: 101–172.

51. Kardong KV (1998) Vertebrates: Comparative anatomy, function, evolution. 2nd edition. Boston: W. C. B. McGraw Hill. 747 p.

52. Williams EE (1959) Gadow' s arcualia and the development of tetrapod vertebrate. Q Rev Biol 34: 1-32.
