# Supplementary material for: A New Saurichthyiform (Actinopterygii) with a Crushing Feeding Mechanism from the Middle Triassic of Guizhou (China)
Source: PLoS One. 2013 Dec 4;8(12):e81010. doi: 10.1371/journal.pone.0081010 (PMC3852010; doi:10.1371/journal.pone.0081010)
Supplement: Text S2 — Data matrix for phylogenetic analysis (Abbreviations Sr .: Saurorhynchus ; Sa .: Saurichthys ; Si .: Sinosaurichthys ). (DOCX) [file pone.0081010.s002.docx]

**Text S2. Data matrix for phylogenetic analysis** (Abbreviations *Sr*.: *Saurorhynchus*; *Sa.*: *Saurichthys; Si*.: *Sinosaurichthys*).

| Taxa | 0000000001 1111111112 2222222223 3333333334 4444444445 5555555556 666666666 |
| --- | --- |
|  | 1234567890 1234567890 1234567890 1234567890 1234567890 1234567890 123456789 |
| *Mimipiscis* | 000?000000 0000000001 001000???0 0000000000 0000000001 0000000000 000100010 |
| *Moythomasia* | 0101000000 1000000002 111000??00 0000000010 0000000000 0000000000 000000000 |
| *Sr.acutus* | 1?0??12111 1111?10002 11?000???2 0100111102 1001000000 ?010?11010 110001130 |
| *Sa.curionii* | ?????13?1? 11????00?? ????0????1 0110111102 1111000000 ?010?11010 100011131 |
| *Si.longipectoralis* | ?????1211? 111??1100? ????0????1 1110111102 1011000000 1010?13111 101011101 |
| *Si.longimedialis* | ?????1211? 111??1100? ????0????1 1110111102 1011000000 1010?13112 101011101 |
| *Sa.ornatus* | 1101111011 1111100002 110000???2 0111111112 1011100000 ?010?01010 100011121 |
| *Sa.madagascariensis* | 1101111011 1111100002 110000???0 0111111112 1011000000 ?010?01010 100001120 |
| *Yelangichthys* | 1100?11011 1120000111 00?111???? ?110011012 ??11111111 1110?020?? ????????? |
| *Birgeria* | ?100?1000? 1100?00?01 00?000???4 0101101010 0000000000 0010?00000 000100010 |
| *Acipenser*  *Amia*  *Australosomus* | 1101110011 1001100000 0000000001 0101101001 102010---0 ---?000000 0000000-0  1110000101 1000000002 1110001110 0100101000 0100101000 0011100000 210100010  0110000001 1000?00002 110010???0 0?00101010 1010100000 0000100000 200100030 |
